# Correction: Targeted modulation of MMP9 and GRP78 via molecular interaction and *in silico* profiling of *Curcuma caesia* rhizome metabolites: A computational drug discovery approach for cancer therapy

**DOI:** 10.1371/journal.pone.0339069

**Published:** 2025-12-15

**Authors:** Mahek Desai, Soham Bhattacharya, Saurabhkumar Mehta, Kaushiki Joshi, Mitesh B. Solanki, Trilok Akhani, Iva Viehmannová, Eloy Fernández Cusimamani

There are errors in the Funding statement. The correct Funding statement is as follows: This work was financially supported by the Internal Grant Agency of the Faculty of Tropical AgriSciences, Czech University of Life Sciences Prague, IGA (Project No. 20253135 and 20253126).
