# Supplementary material for: Targeted modulation of MMP9 and GRP78 via molecular interaction and in silico profiling of Curcuma caesia rhizome metabolites: A computational drug discovery approach for cancer therapy
Source: PLoS One. 2025 Jul 18;20(7):e0328509. doi: 10.1371/journal.pone.0328509 (PMC12273913; doi:10.1371/journal.pone.0328509)
Supplement: S1 Table — (PDF) [file pone.0328509.s001.pdf]

S1 Table: Phytochemical dataset of *C. caesia* rhizome

Compound Name	PubChem ID	References
Bis-demethoxycurcumin	5315472	(Ibrahim et al., 2023)
Curcumin	969516	(Ibrahim et al., 2023)
Ar-turmerone	160512	(Ibrahim et al., 2023; Gangal et al., 2023)
Xanthorrhizol	93135	(Gangal et al., 2023)
Ar-Curcumene	3083834	(Ibrahim et al., 2023; Gangal et al., 2023)
Chavicol methyl	70235324	(Mahanta et al., 2022)
5, 8, 11, 14, 17 – eicosapentaenoic acid	446284	(Mahanta et al., 2022)
Gamma-Curcumene	12304273	(Mahanta et al., 2022)
Farnesol	3327	(Mahanta et al., 2022)
Retinal	638015	(Mahanta et al., 2022)
Zingiberene	92776	(Mahanta et al., 2022)
Beta-sitosterol	222284	(Ibrahim et al., 2023)
1, 8, 15, 22-Tricosatetrayne	138309	(Gangal et al., 2023)
Benzene, 1-(1, 5-dimethyl-1, 4-hexenyl)-4-methyl	577053	(Mahanta et al., 2022)
Boldenone	13308	(Mahanta et al., 2022)
Dihydrocarveol	12072	(Mahanta et al., 2022)
Alpha-bulnesene	94275	(Ibrahim et al., 2023; Gangal et al., 2023)
Androstenediol	10634	(Mahanta et al., 2022)
Curdione	5362828	(Gangal et al., 2023)
p-cymene	7463	(Mahanta et al., 2022)
Tricyclo[8.6.0.0(2, 9)]hexadeca-3, 15-diene	91697587	(Mahanta et al., 2022)
Alpha-terpineol	17100	(Mahanta et al., 2022)
Delta-cadinene	441005	(Gangal et al., 2023)
(z, e)-alpha-farnasene	5362889	(Mahanta et al., 2022)
4-dimethylaminobenzoic acid	12092	(Mahanta et al., 2022)
Limonene	440917	(Mahanta et al., 2022)
Terpinolene	11463	(Mahanta et al., 2022)
Carabrone	164879	(Mahanta et al., 2022)

Estragole	8815	(Mahanta et al., 2022)
Germacrene B	5281519	(Gangal et al., 2023)
Beta-cubebene	93081	(Mahanta et al., 2022)
6-isopropenyl-4, 8a-dimethyl-1, 2, 3, 5, 6, 7, 8, 8a-octahydronaphthalene-2ol	594234	(Mahanta et al., 2022)
Intermedeol	1556033	(Gangal et al., 2023)
Spathulenol	92231	(Mahanta et al., 2022)
2, 7-dimethyl oxepine	578868	(Mahanta et al., 2022)
Curcumenol	167812	(Gangal et al., 2023)
Furanodienone	6506548	(Mahanta et al., 2022)
Beta-selinene	28237	(Mahanta et al., 2022)
Gamma-cadinene	6432404	(Mahanta et al., 2022)
(z)-beta-ocimene	5320250	(Ibrahim et al., 2023; Gangal et al., 2023)
Iso-Curcumenol	10399139	(Gangal et al., 2023)
Ledol	92812	(Ibrahim et al., 2023; Gangal et al., 2023)
Linalool	6549	(Mahanta et al., 2022)
Occidentalol	1138655	(Mahanta et al., 2022)
Rosifoliol	527256	(Mahanta et al., 2022)
Alpha-santalol	11085337	(Mahanta et al., 2022)
Alpha-terpinolene	11463	(Mahanta et al., 2022)
(1, 1, 4, 4-tetramethyl-2, 3-tetralindione)	512443	(Mahanta et al., 2022)
1, 8-cineole (Eucalyptol)	2758	(Ibrahim et al., 2023; Gangal et al., 2023)
Curzerenone	3081930	(Gangal et al., 2023)
Furanodiene	9601230	(Mahanta et al., 2022)
Tropolone	10789	(Ibrahim et al., 2023; Gangal et al., 2023)
Alpha-cadinol	519662	(Mahanta et al., 2022)
(-)-Neoclovene-(I), dihydro-	557156	(Gangal et al., 2023)
Alloaromadendrene	10899740	(Mahanta et al., 2022)
Caryophyllene oxide	1742210	(Mahanta et al., 2022)
Cis-alpha-copaene-8-ol	25086830	(Mahanta et al., 2022)
Terpinen-4-ol	2724161	(Mahanta et al., 2022)

(E, E)-Germacrone	6436348	(Gangal et al., 2023)
7-Epi-Alpha-eudesmol	12304196	(Mahanta et al., 2022)
Bicyclo[3.1.0]hexane, 6-isopropylidene	556529	(Mahanta et al., 2022)
Elemane	12309447	(Mahanta et al., 2022)
Epiglobulol	11858788	(Mahanta et al., 2022)
Germacrane	9548707	(Mahanta et al., 2022)
Germacrene D	5317570	(Gangal et al., 2023)
Germacrone	6436348	(Ibrahim et al., 2023)
Trans-sesquisabinene hydrate	6428444	(Mahanta et al., 2022)
Beta-eudesmol	91457	(Mahanta et al., 2022)
Globulol	12304985	(Mahanta et al., 2022)
Beta-guaiene	15560252	(Mahanta et al., 2022)
5-nonanone	10405	(Mahanta et al., 2022)
Viridiflorol	11996452	(Mahanta et al., 2022)
Alpha-selinene	10856614	(Mahanta et al., 2022)
Beta-Caryophyllene	20831623	(Mahanta et al., 2022)
Caryophyllene	5281515	(Ibrahim et al., 2023)
Delta-Elemene	12309449	(Gangal et al., 2023)
Beta-elemene	6918391	(Ibrahim et al., 2023; Gangal et al., 2023)
Megastigmatrienone	5375190	(Mahanta et al., 2022)
(+)-2-bornanone	9543187	(Mahanta et al., 2022)
Elemol	92138	(Mahanta et al., 2022)
Menthone	26447	(Mahanta et al., 2022)
Beta-elemenone	10955018	(Ibrahim et al., 2023; Gangal et al., 2023)
Gamma-elemene	6432312	(Gangal et al., 2023)
Myrcene	31253	(Mahanta et al., 2022)
Beta-pinene	14896	(Gangal et al., 2023)
Bornyl acetate	6448	(Ibrahim et al., 2023; Gangal et al., 2023)
Megastigma-3, 7(e), 9-triene	5369743	(Mahanta et al., 2022)
Alpha-pinene	6654	(Gangal et al., 2023)
2-cyclohexen-1-one	13594	(Mahanta et al., 2022)
Bicyclo[3.1.0]hexane-3-one	549318	(Mahanta et al., 2022)

4-(dimethylamino)-3, 5-dimethyl-phenol	21102	(Mahanta et al., 2022)
Camphor	2537	(Ibrahim et al., 2023; Gangal et al., 2023)
Cyclohexanol	7966	(Mahanta et al., 2022)
Isoborneol	6321401	(Gangal et al., 2023)
Isomenthone	70962	(Mahanta et al., 2022)
Delta-3-carene	442461	(Mahanta et al., 2022)
Borneol	6552009	(Ibrahim et al., 2023; Gangal et al., 2023)
Camphene	6616	(Gangal et al., 2023)
Endo-fenchol	15406	(Mahanta et al., 2022)
Camphene hydrate	101680	(Mahanta et al., 2022)
Benzene	241	(Ibrahim et al., 2023)

References:

1. Ibrahim NNA, Wan Mustapha WA, Sofian-Seng NS, Lim SJ, Mohd Razali NS, Teh AH, et al. A Comprehensive Review with Future Prospects on the Medicinal Properties and Biological Activities of *Curcuma caesia* Roxb . Russo D, editor. Evid Based Complement Alternat Med. 2023 Jan;2023(Ibrahim et al., 2023):7006565.

2. Gangal A, Duseja M, Sethiya NK. Chemical composition, in vitro antioxidant and α-amylase inhibitory activities of rhizomes essential oil and nutrient components from rhizomes powder of Curcuma caesia Roxb. (black turmeric) collected from Garhwal region of Uttrakhand, India. J Essent Oil Bear Plants. 2023 Nov 2;26(6):1473–86.

3. Mahanta BP, Kemprai P, Bora PK, Lal M, Haldar S. Phytotoxic essential oil from black turmeric (*Curcuma caesia* Roxb.) rhizome: Screening, efficacy, chemical basis, uptake and mode of transport. Ind Crops Prod. 2022 Jun 1;180:114788.
