# Supplementary material for: Targeted modulation of MMP9 and GRP78 via molecular interaction and in silico profiling of Curcuma caesia rhizome metabolites: A computational drug discovery approach for cancer therapy
Source: PLoS One. 2025 Jul 18;20(7):e0328509. doi: 10.1371/journal.pone.0328509 (PMC12273913; doi:10.1371/journal.pone.0328509)
Supplement: S3 Table — (PDF) [file pone.0328509.s003.pdf]

S3 Table: Binding free-energy values (ΔG in kcal/mol) and interactions of selected *C. caesia* rhizome metabolites with GRP78 and MMP9

Compound Name	PDB ID: 5F1X		PDB ID: 1GKC	
	**Binding Affinity**	**Interaction**	**Binding Affinity**	**Interaction**
Bis-demethoxycurcumin	-8.0	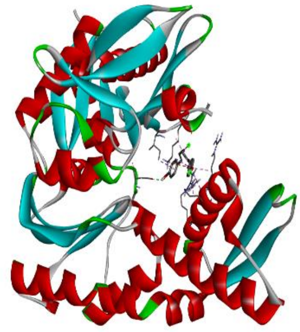	-9.1	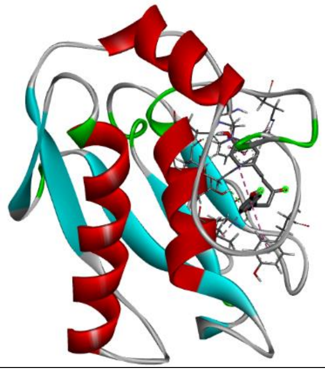
Curcumin	-8.5	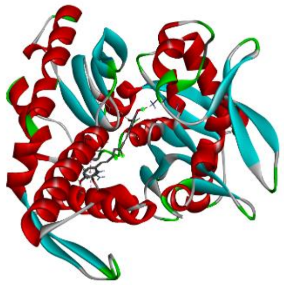	-8.0	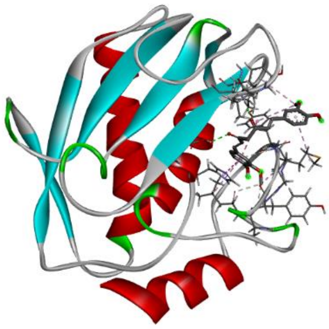
ar-turmerone	-7.4	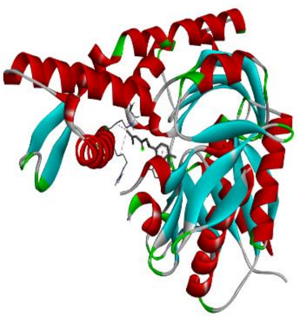	-7.9	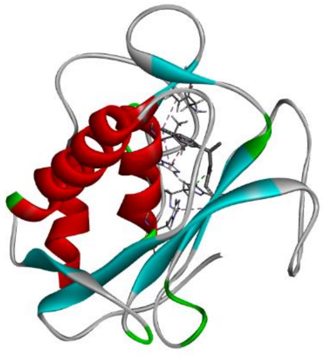
Xanthorrhizol	-7.3	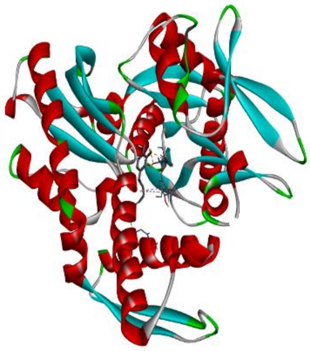	-7.8	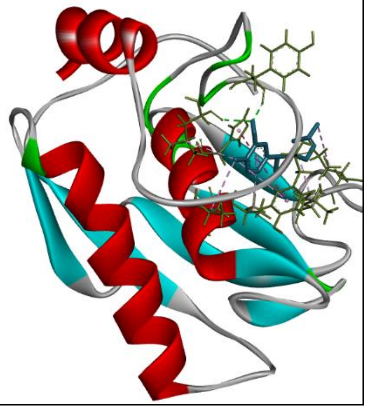

ar-Curcumene	-7.1	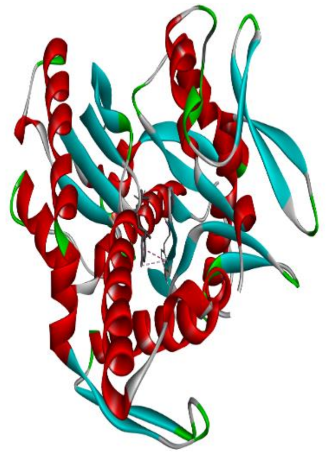	-7.5	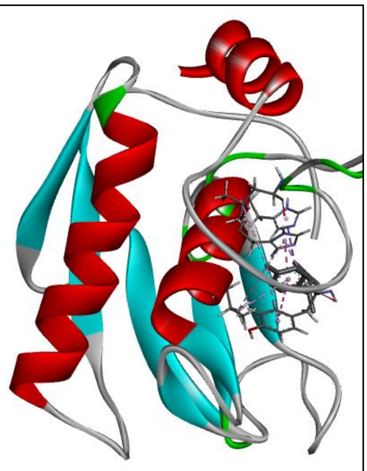
Chavicol methyl	-6.3	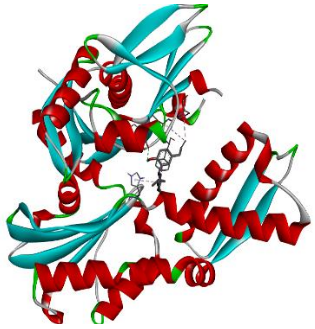	-7.5	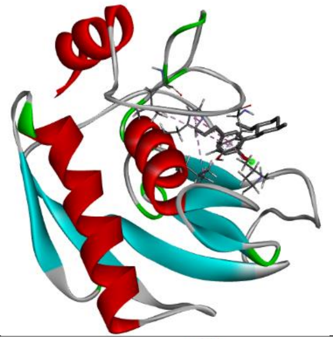
5,8,11,14,17-eicosapentaenoic acid	-6.5	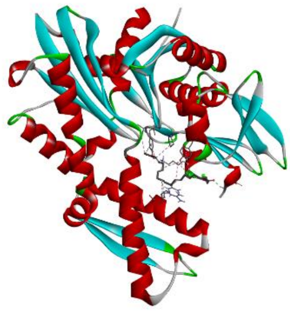	-7.4	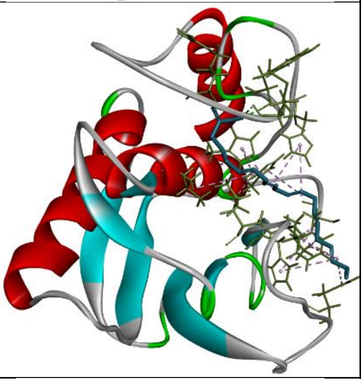
Gamma-Curcumene	-7.0	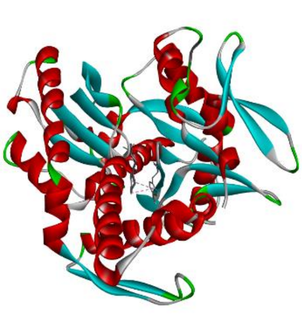	-7.4	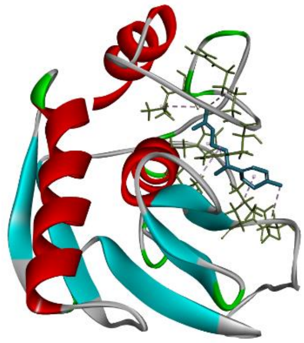

Farnesol	-6.1	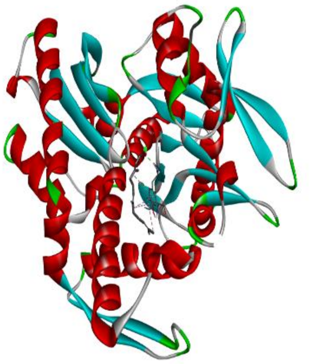	-7.3	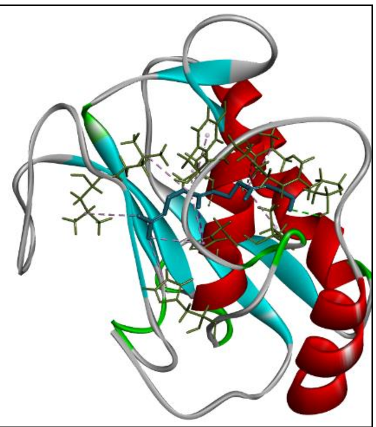
Retinal	-7.2	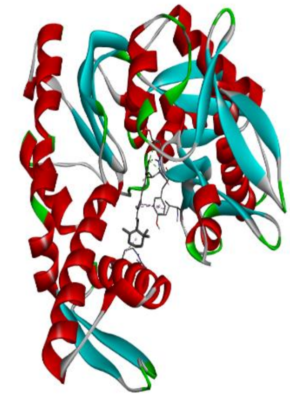	-7.3	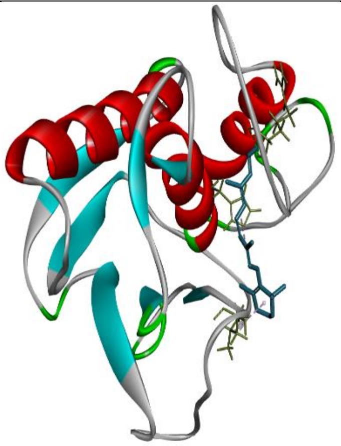
Zingiberene	-7.0	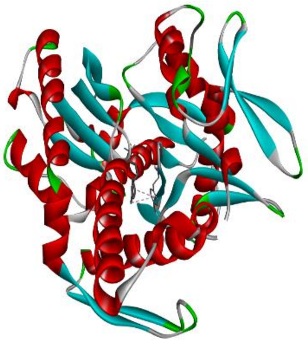	-7.3	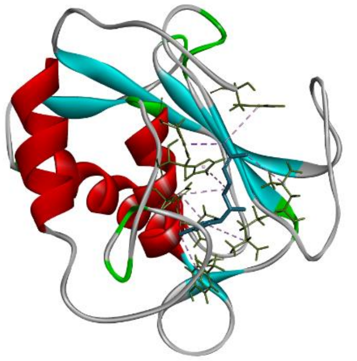
Beta-sitosterol	-8.1	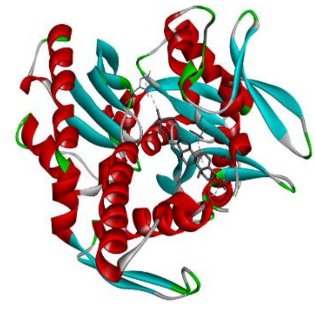	-7.3	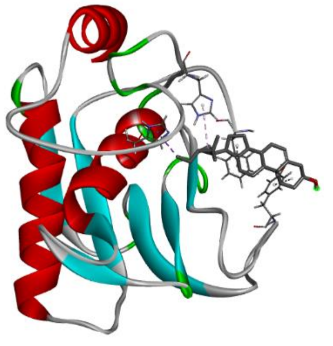

1,8,15,22-Tricosatetrayn	-4.5	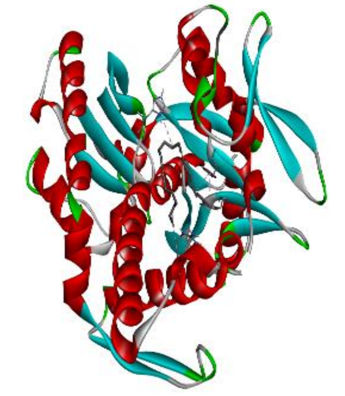	-7.2	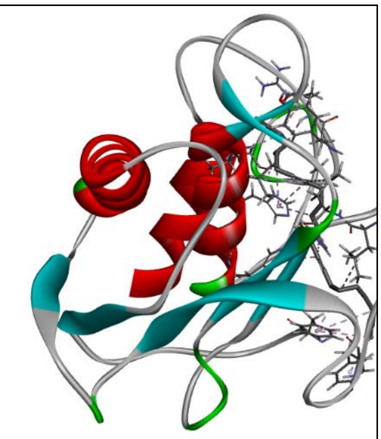
Benzene,1-(1,5-dimethyl-4-hexenyl)- 4-methyl	-5.9	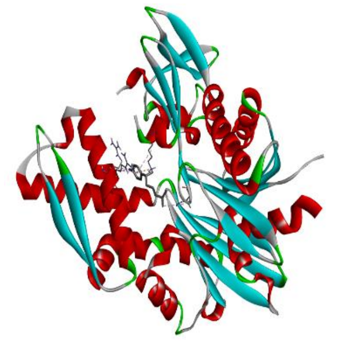	-7.2	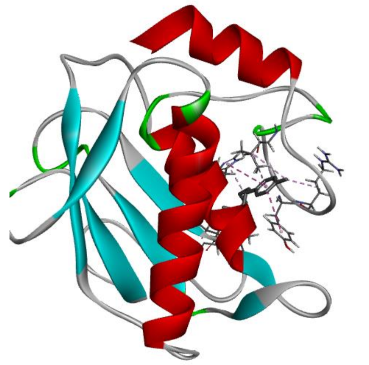
Boldenone	-7.6	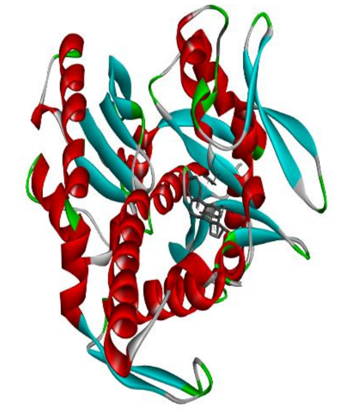	-7.2	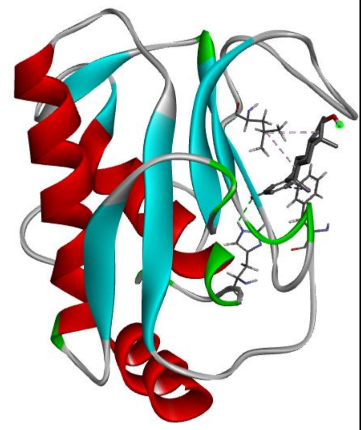
Dihydrocarveol	-6.2	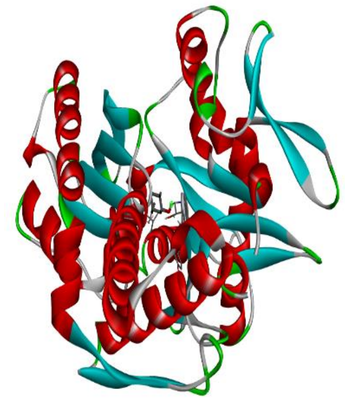	-7.1	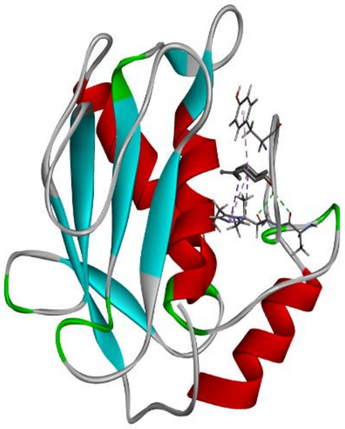

Alpha-bulnesene	-7.6	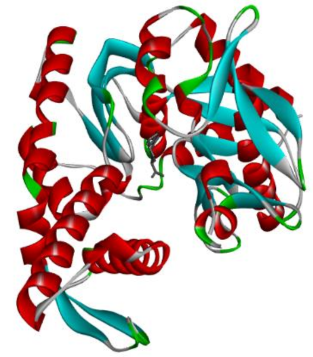	-7.1	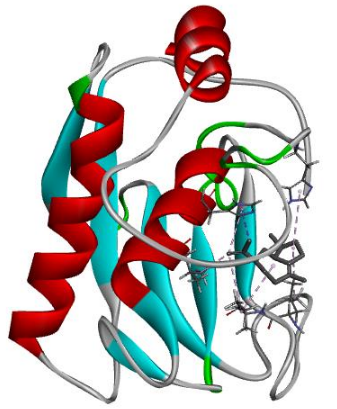
Androstenediol	-8.0	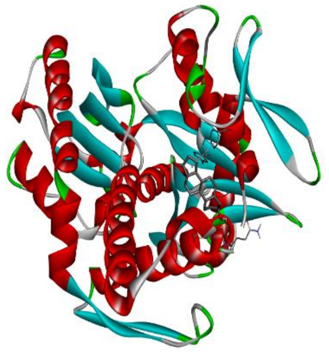	-6.9	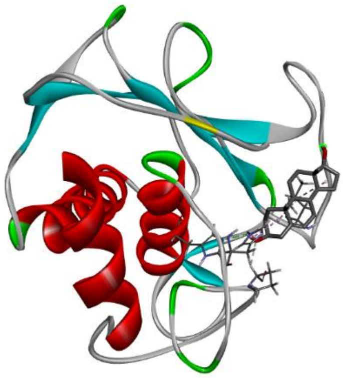
Curdione	-6.7	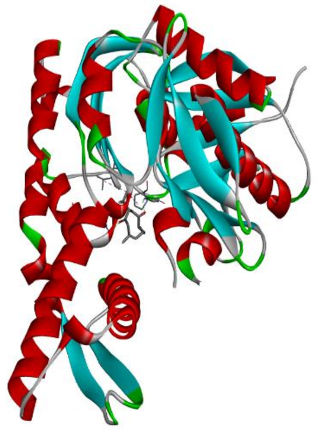	-6.8	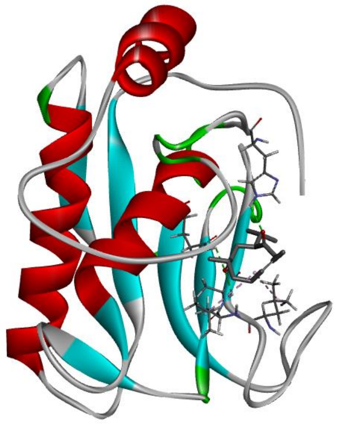
p-cymene	-5.6	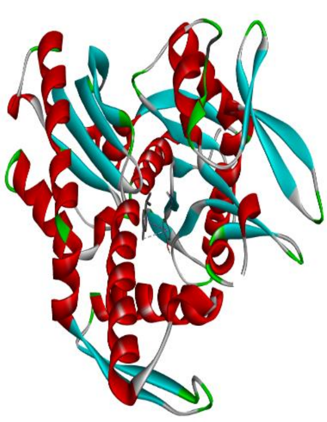	-6.8	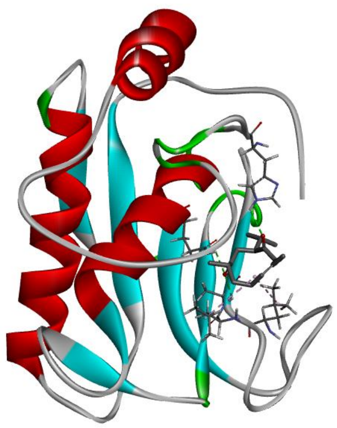

Tricyclo-[8.6.0.0(2,9)]hexadeca-3,15-diene	-8.2	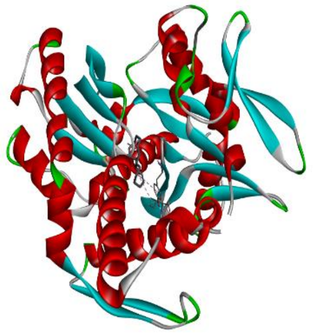	-6.8	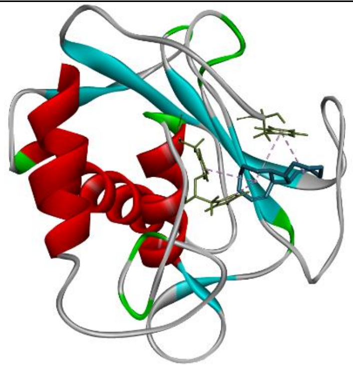
Alpha-terpineol	-5.9	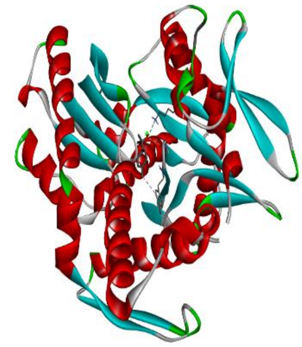	-6.8	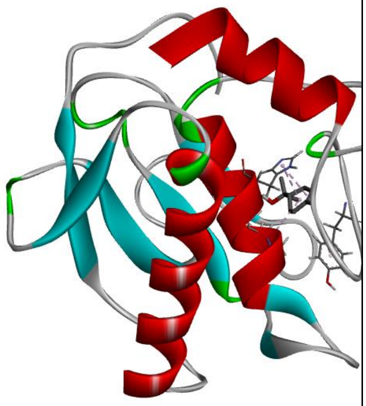
Delta-cadinene	-7.3	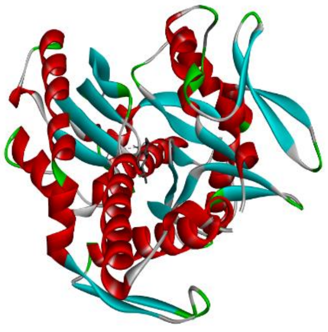	-6.7	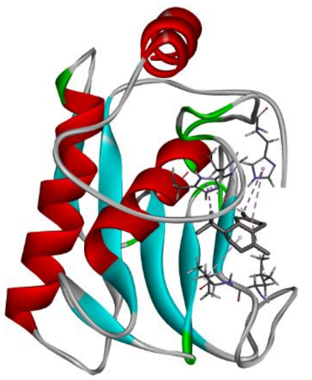
(z,e)-alpha-farnesene	-6.3	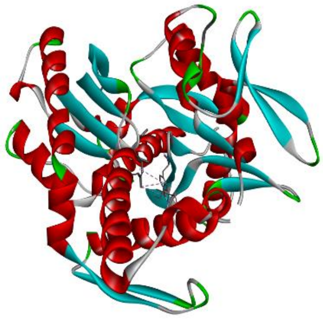	-6.6	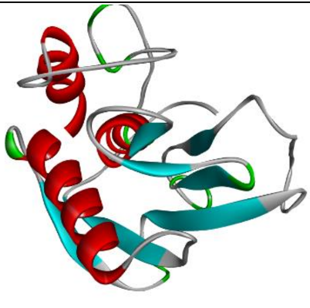
4-dimethyamino-benzoic acid	-6.2	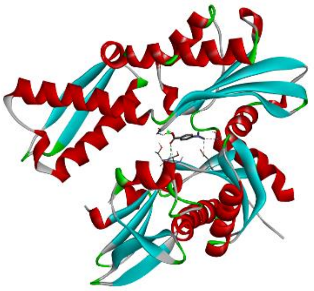	-6.6	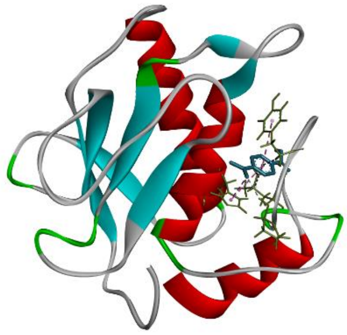

Limonene	-5.5	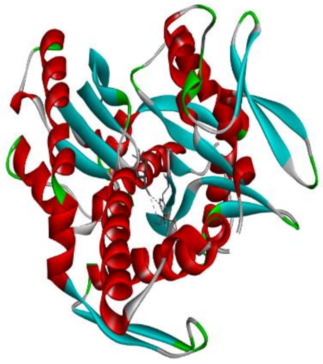	-6.6	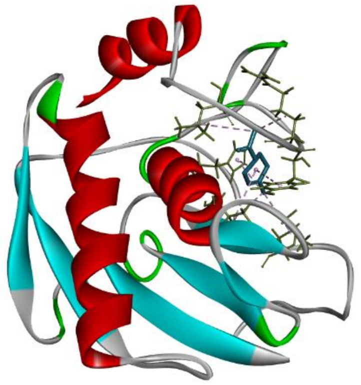
Terpinolene	-5.6	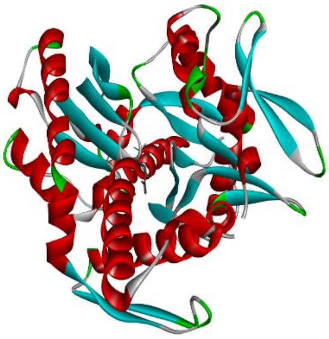	-6.6	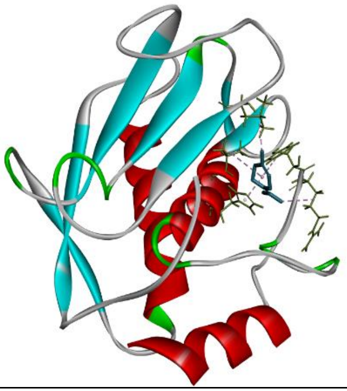
Carabrone	-6.7	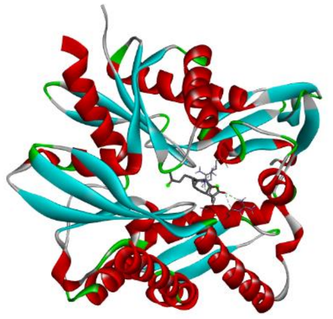	-6.4	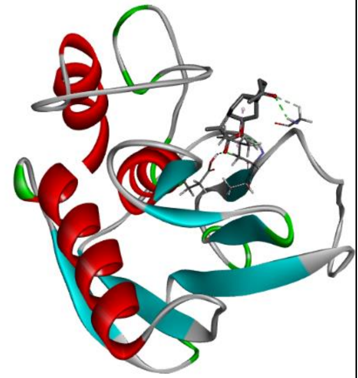
Estragole	-5.6	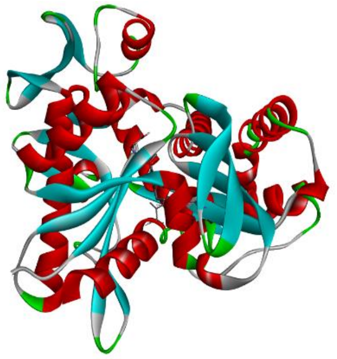	-6.4	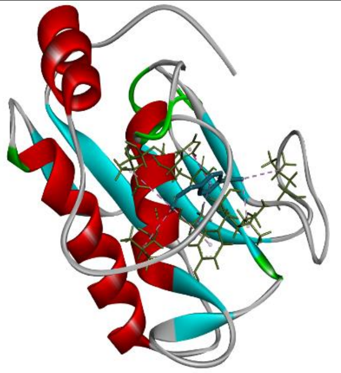

Germacrene b	-6.1	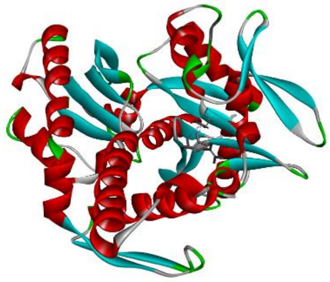	-6.4	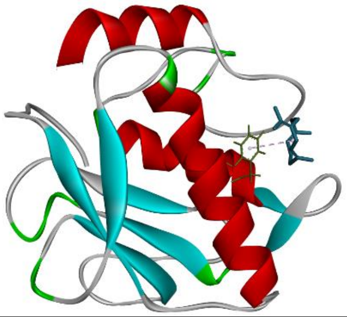
Beta-cubebene	-6.5	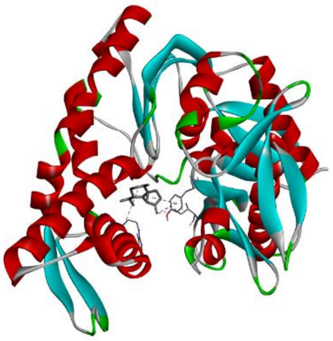	-6.4	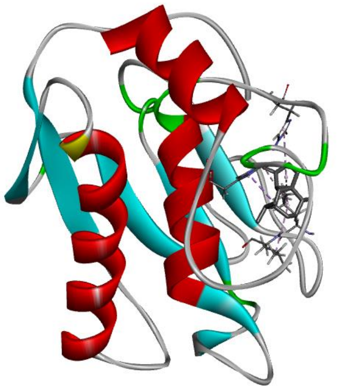
6-isopropenyl-4,8a-dimethyl-1,2,3,5,6,7,8,8a-octahydro-napthalene-2ol	-8.1	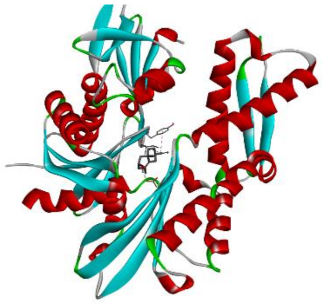	-6.3	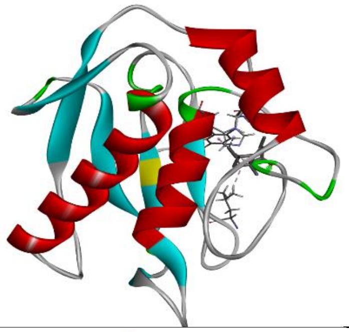
Intermedeol	-6.4	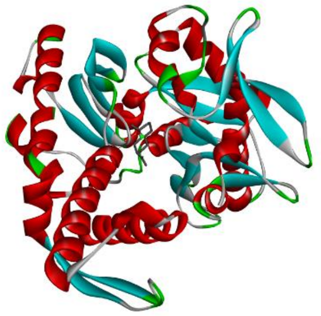	-6.3	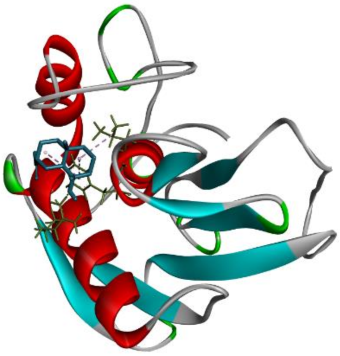
Spathulenol	-7.2	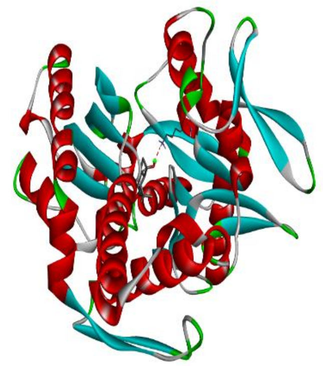	-6.3	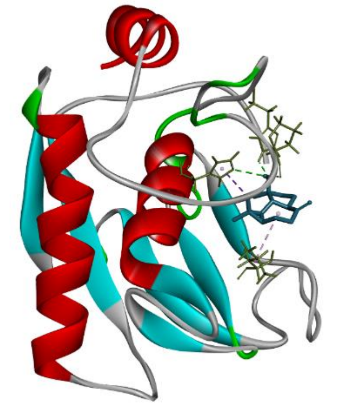

2,7-dimethyloxepine	-5.1	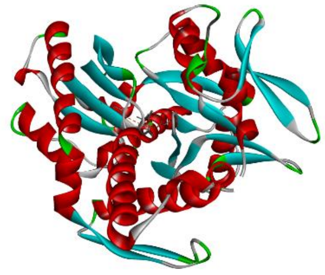	-6.2	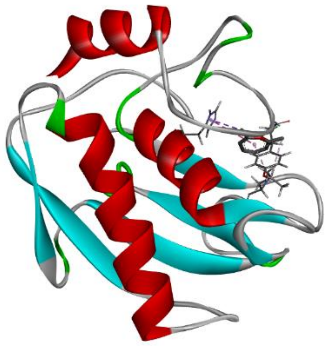
Curcumenol	-6.4	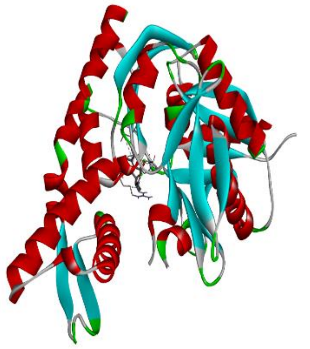	-6.2	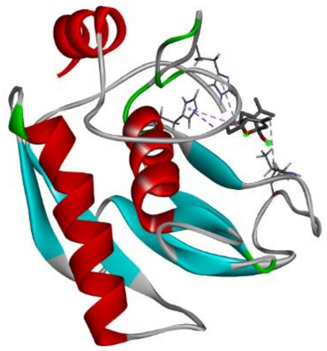
Furanodienone	-7.0	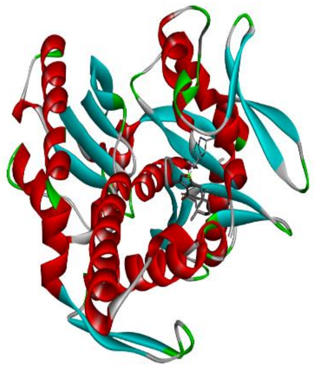	-6.2	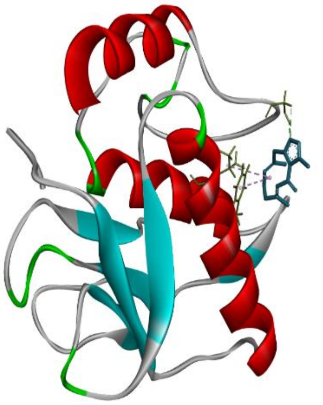
Beta-selinene	-7.1	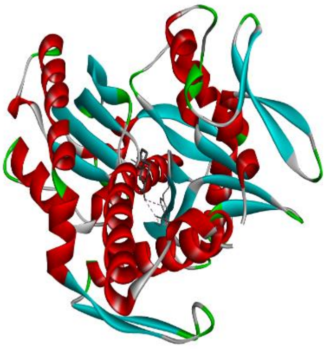	-6.2	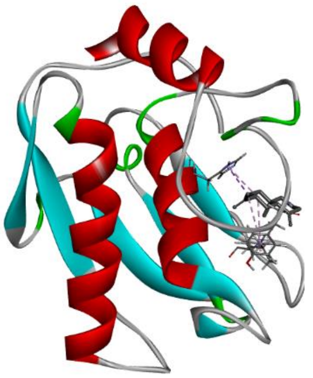

Gamma-cadinene	-6.3	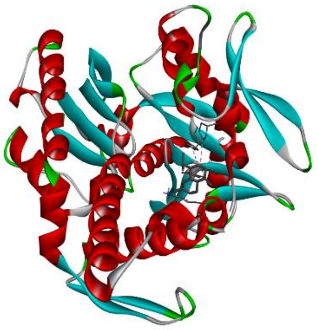	-6.2	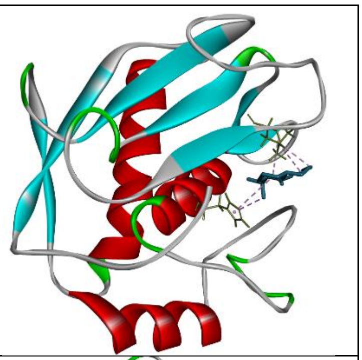
(z)-beta-ocimene	-5.1	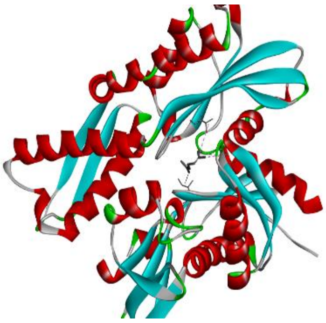	-6.1	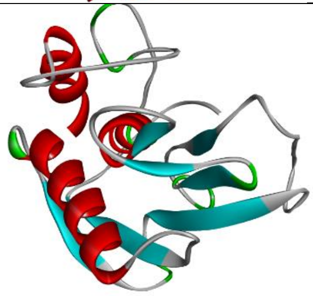
Iso-Curcumenol	-6.9	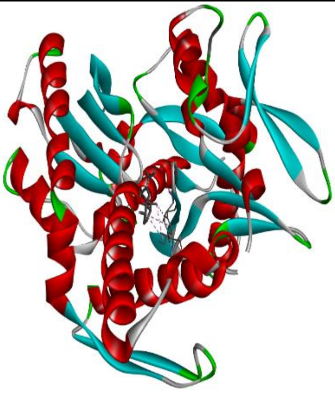	-6.1	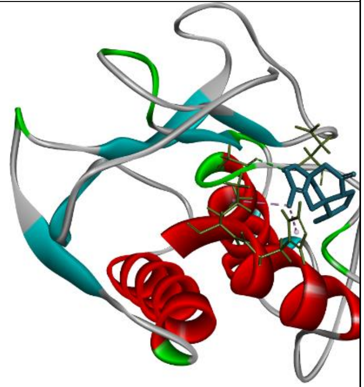
Ledol	-6.3	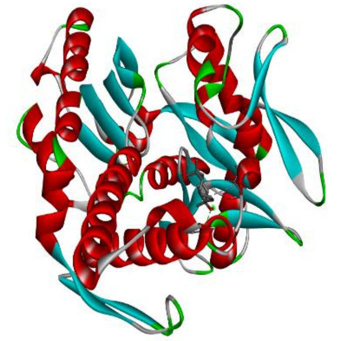	-6.1	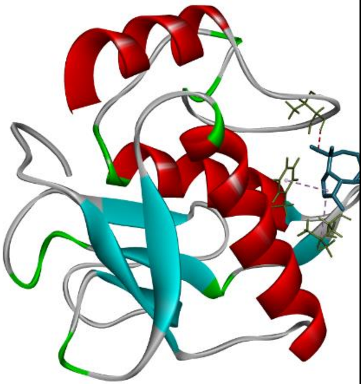
Linalool	-5.3	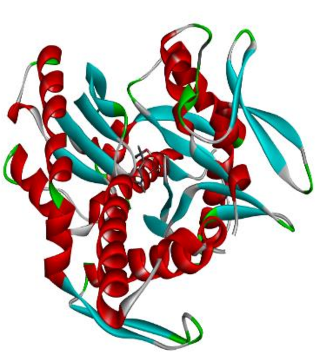	-6.1	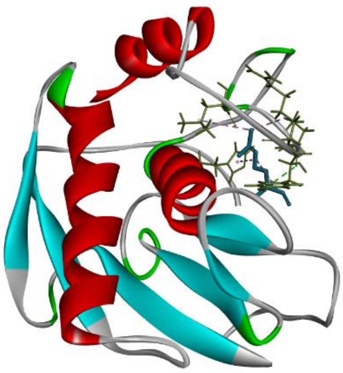

Occidentalol	-6.4	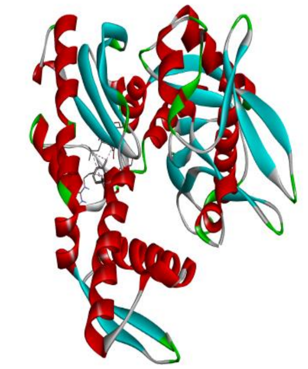	-6.1	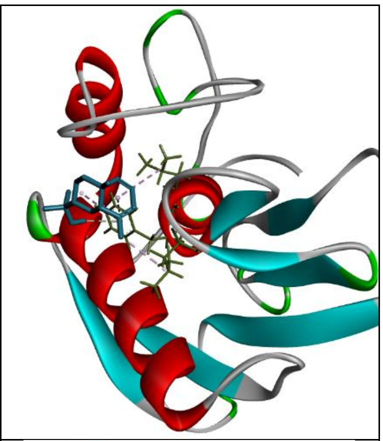
Rosifoliol	-6.3	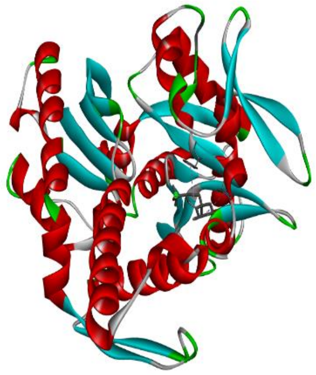	-6.1	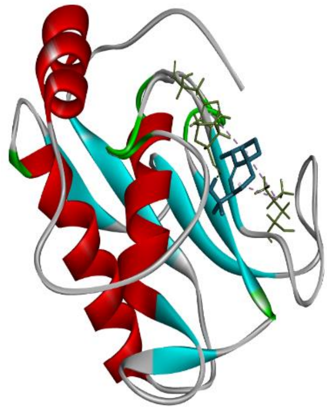
Alpha-santalol	-5.9	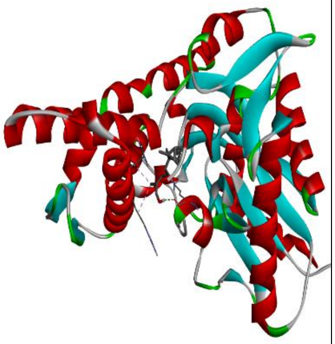	-6.1	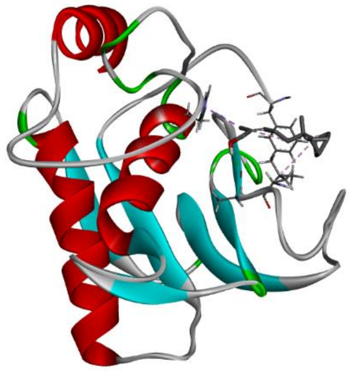
Alpha-terpinolene	-5.6	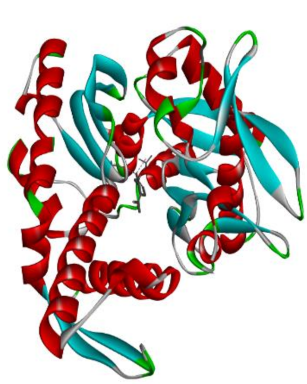	-6.1	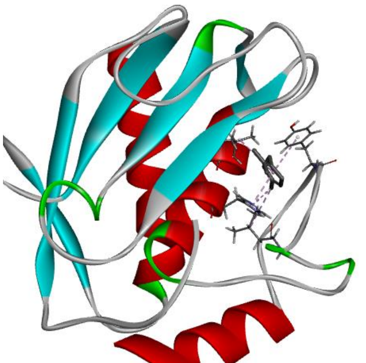

(1,1,4,4-tetramethyl-2,3-tetralindione)	-6.6	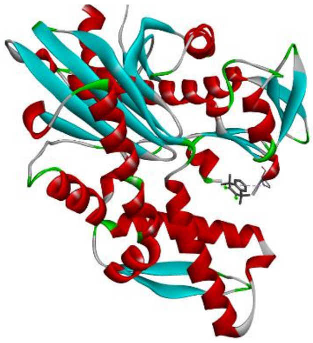	-6.0	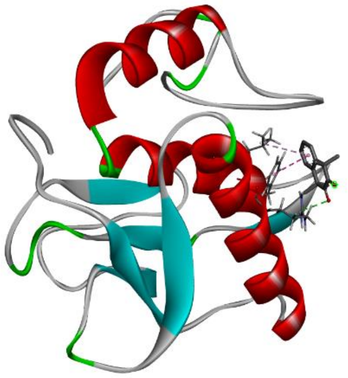
1,8-cineole (Eucalyptol)	-5.6	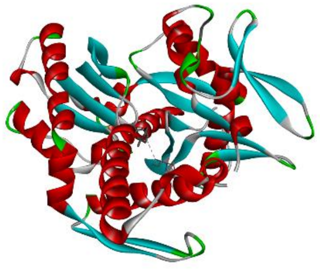	-6.0	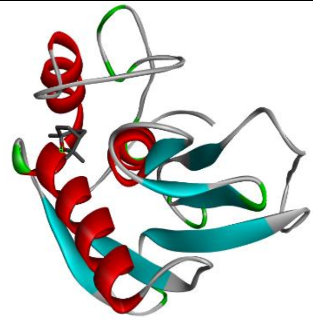
Curzerenone	-6.3	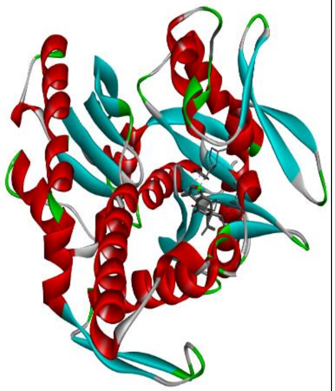	-6.0	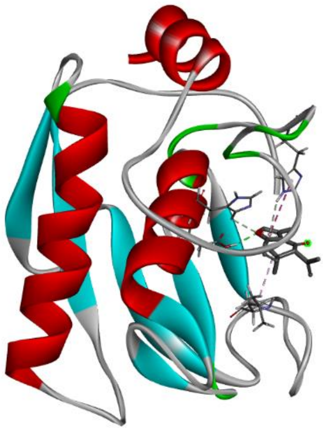
Furanodiene	-6.6	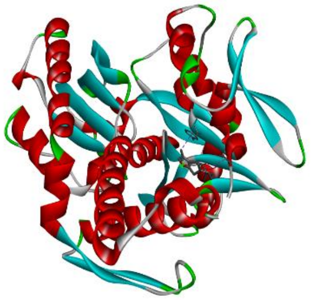	-6.0	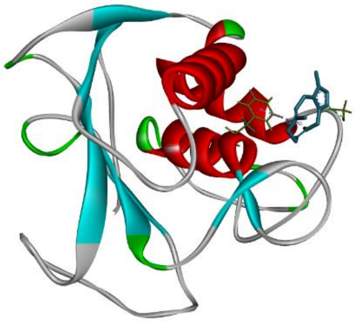
Tropolone	-5.6	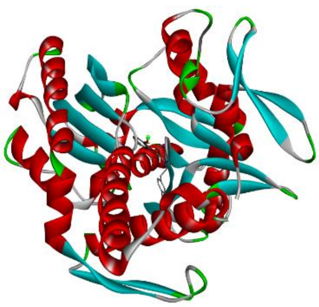	-6.0	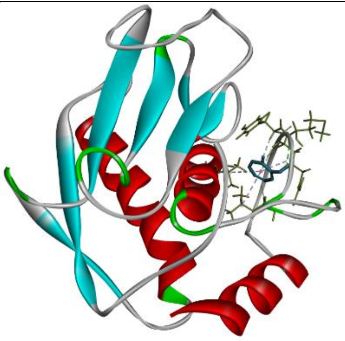

Alpha-cadinol	-6.1	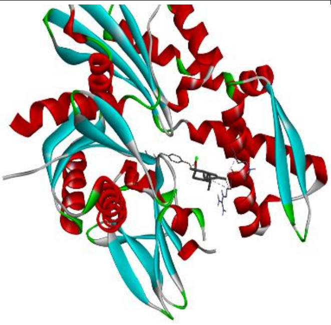	-6.0	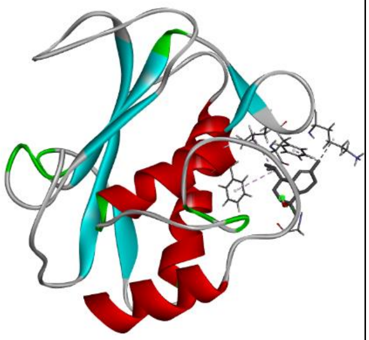
(-)-Neoclovene-(I), dihydro-cid	-7.2	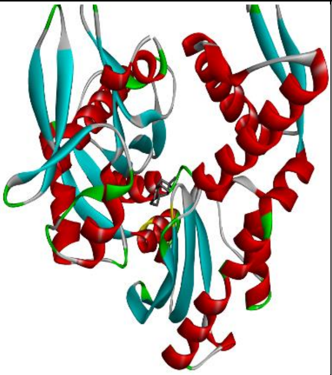	-5.9	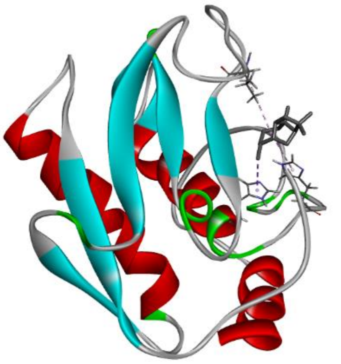
Alloaromadendrene	-6.0	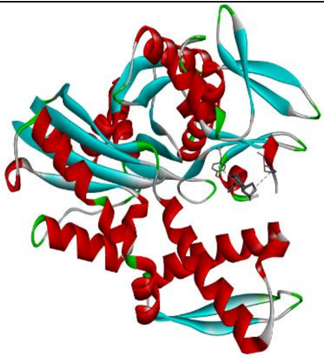	-5.9	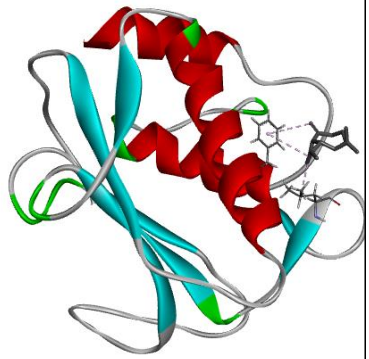
Cayrophyllene oxide	-6.4	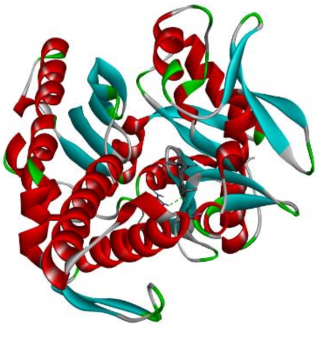	-5.9	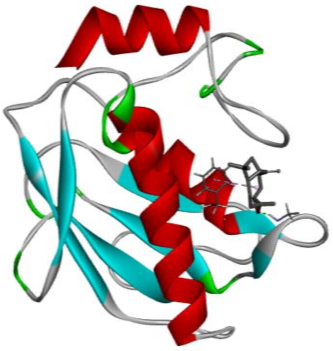
cis-alpha-copaene-8-ol	-7.2	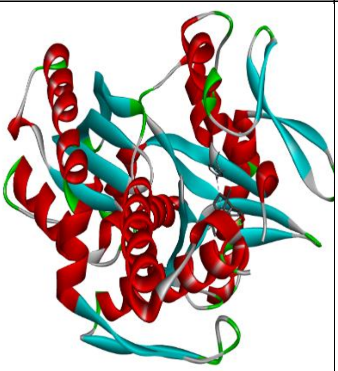	-5.9	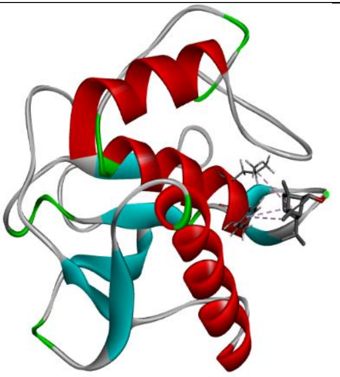

Terpinen-4-ol	-6.0	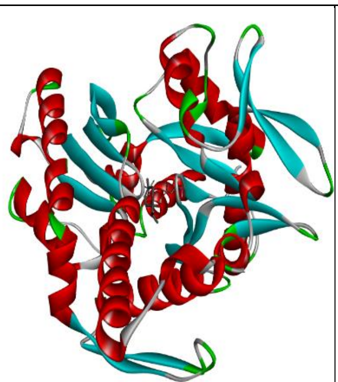	-5.9	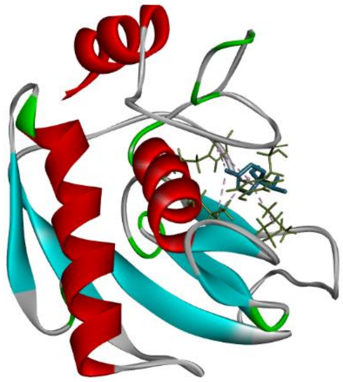
(E, E)-Germacrone	-6.3	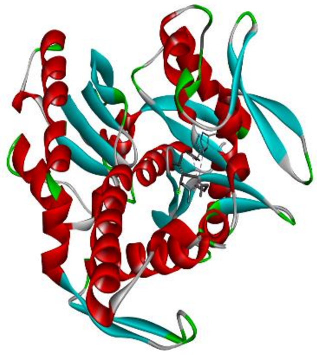	-5.8	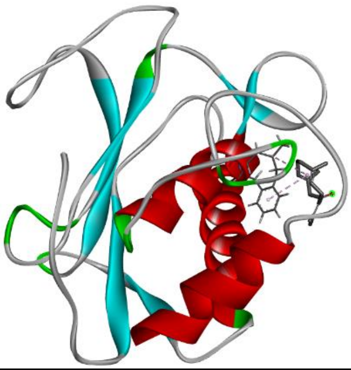
7-Epi-Alpha-eudesmol	-7.2	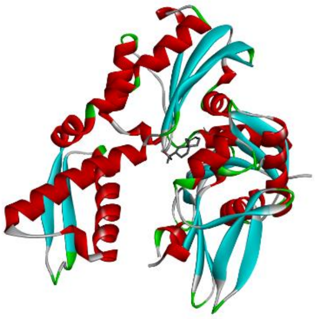	-5.8	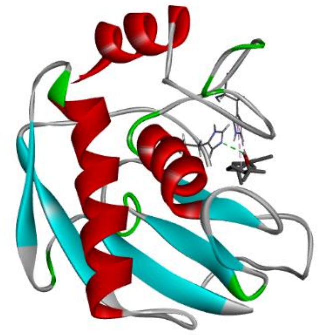
Bicyclo[3.1.0]hexane, 6-isopropylidene-	-5.1	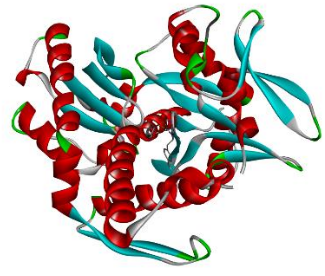	-5.6	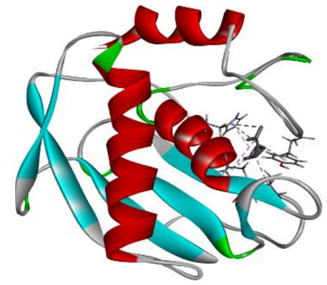
Elemane	-5.8	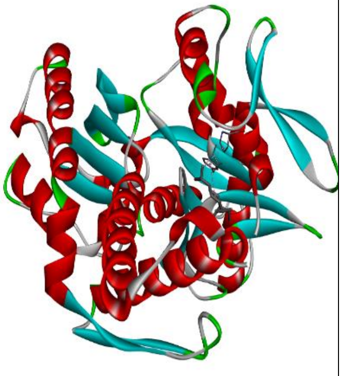	-5.8	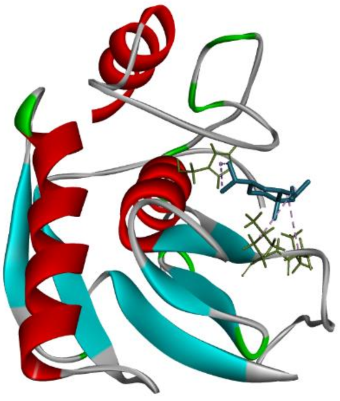

Epiglobulol	-7.4	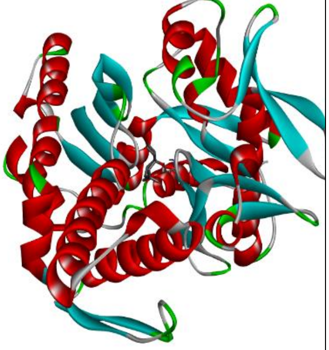	-5.8	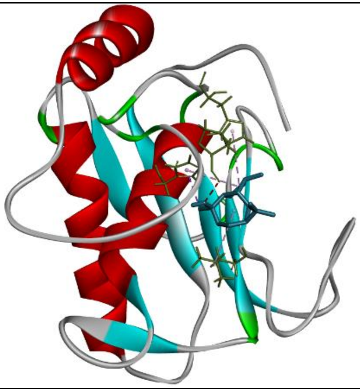
Germacrane	-6.1	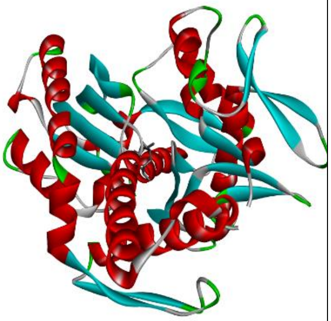	-5.8	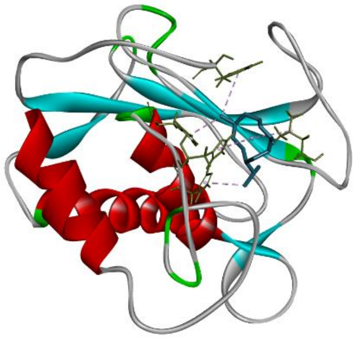
Germacrene D	-6.3	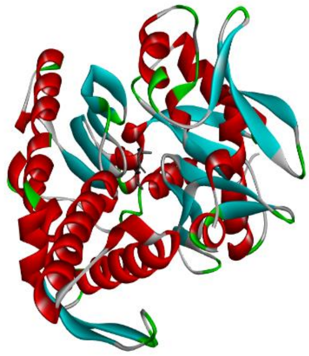	-5.8	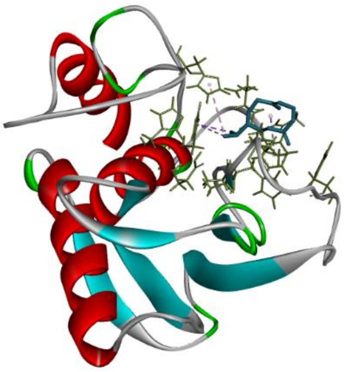
Germacrone	-6.3	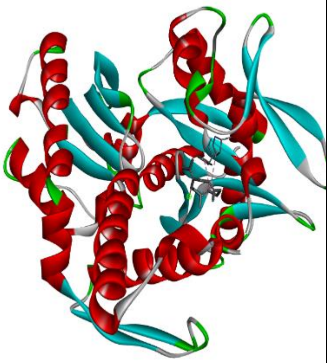	-5.8	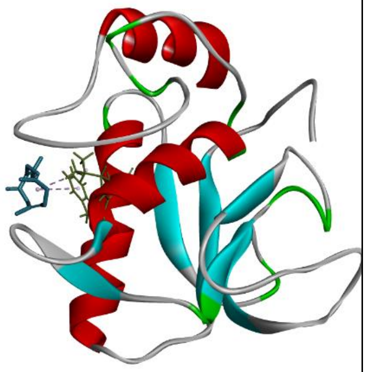
Trans-sesquisabinene hydrate	-5.5	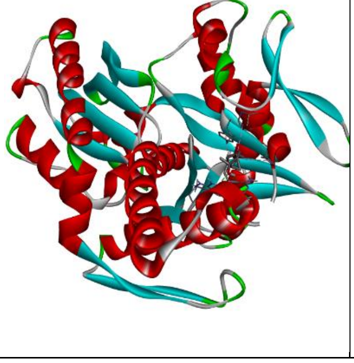	-5.8	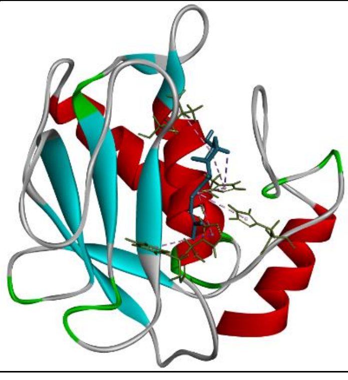

Beta-eudesmol	-7.0	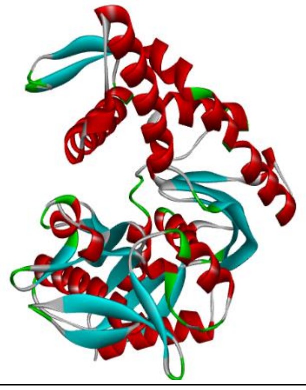	-5.8	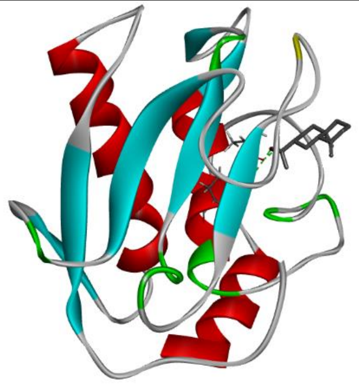
Globulol	-7.8	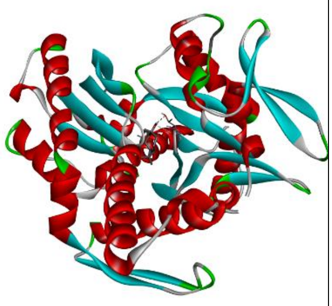	-5.7	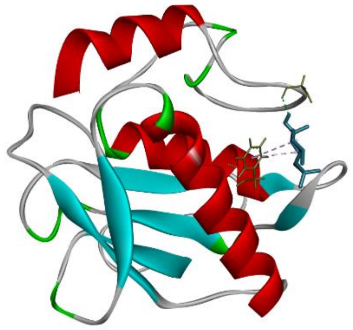
Beta-guaiene	-5.9	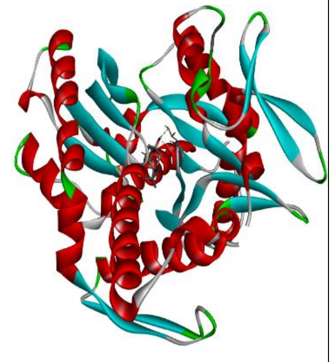	-5.7	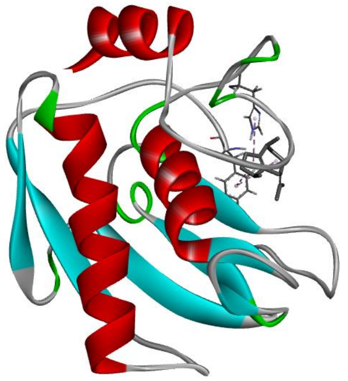
5-nonanone	-4.2	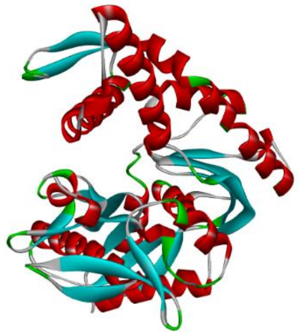	-5.6	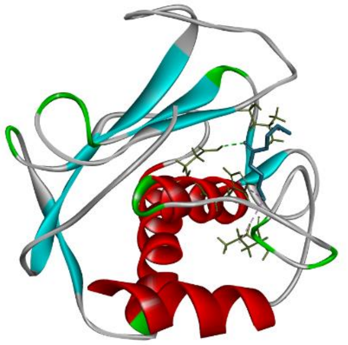

Viridiflorol	-8.1	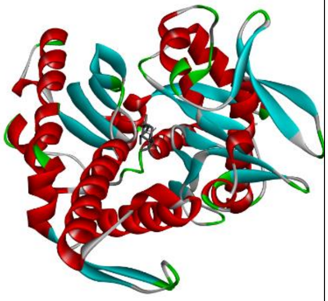	-5.6	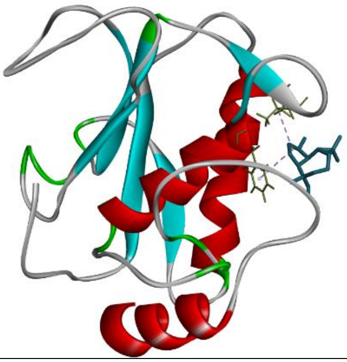
Alpha-selinene	-6.2	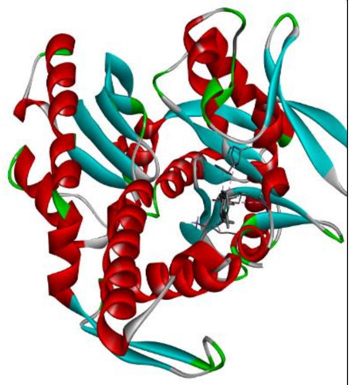	-5.6	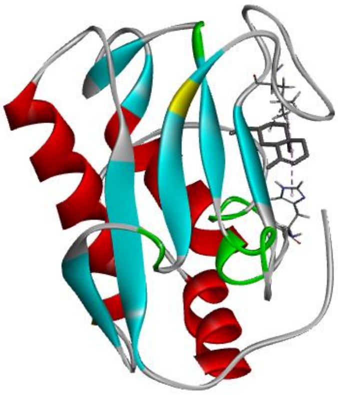
Beta-Caryophyllene	-6.1	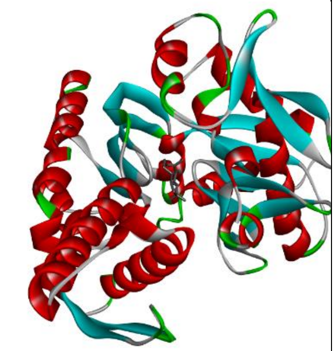	-5.5	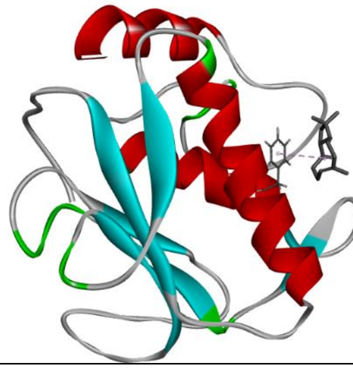
Caryophyllene	-5.9	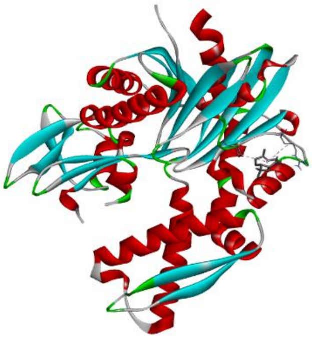	-5.5	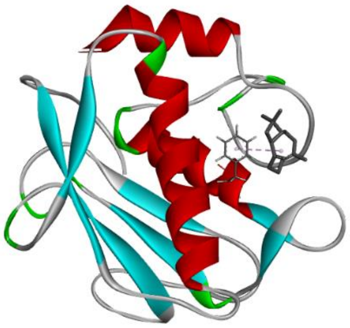

Delta-Elemene	-6.1	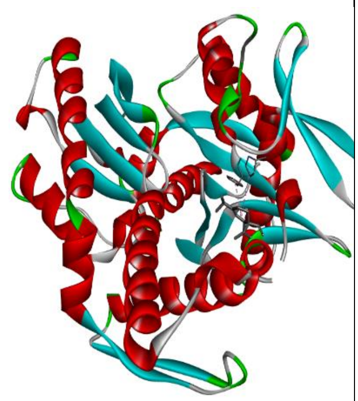	-5.5	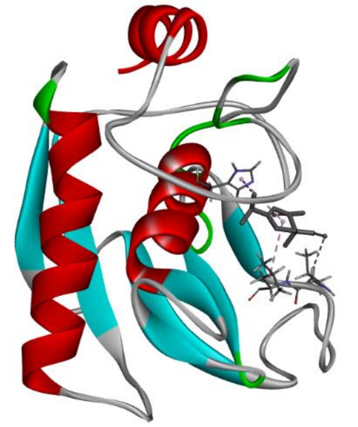
Beta-elemene	-6.6	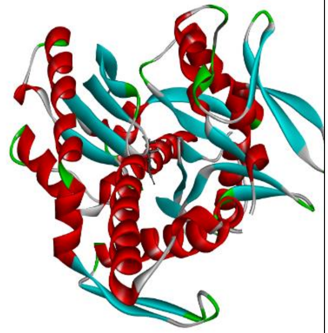	-5.4	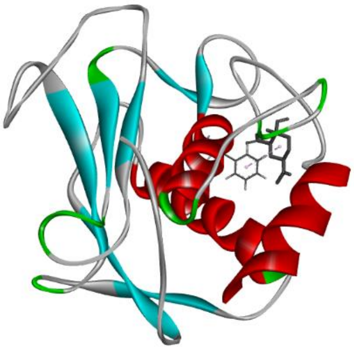
Megastigmatrienone	-6.3	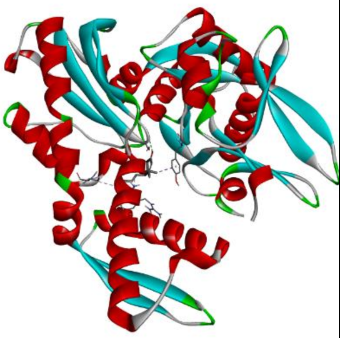	-5.4	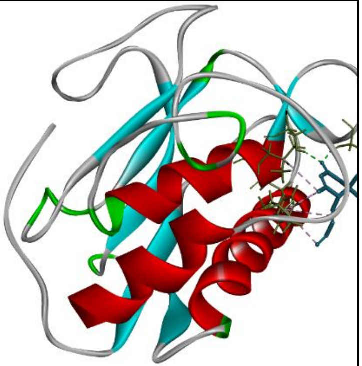
(+)-2-bornanone	-5.8	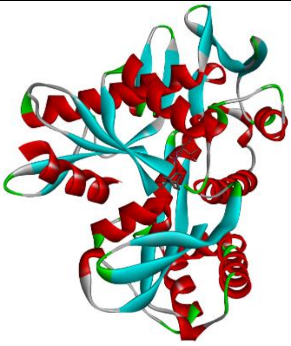	-5.3	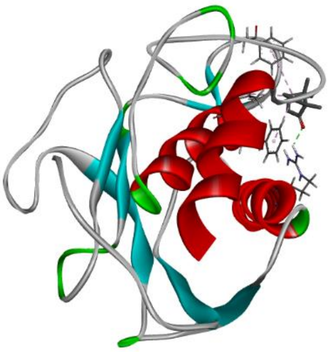

Elemol	-6.2	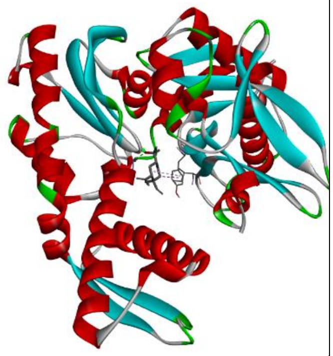	-5.3	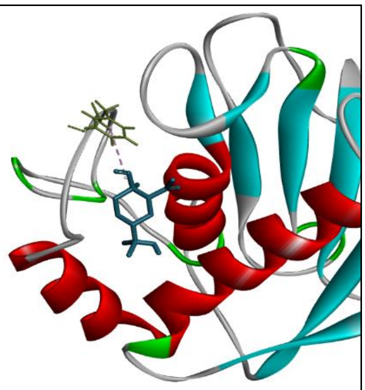
Menthone	-5.8	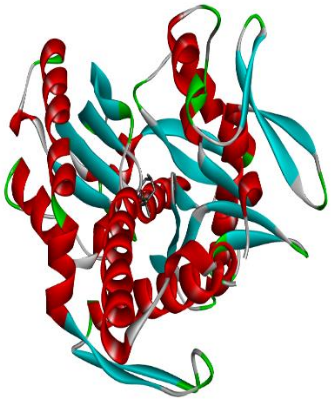	-5.3	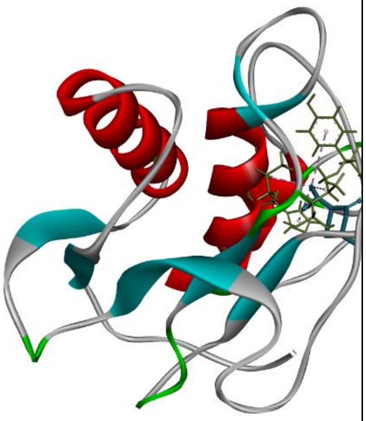
Beta-elemenone	-7.0	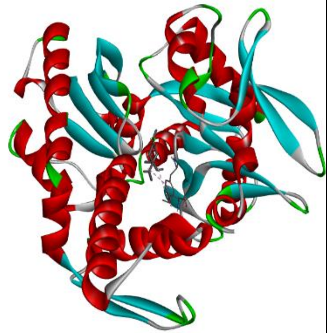	-5.3	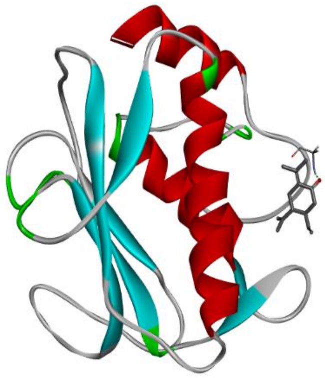
Gamma-elemene	-5.9	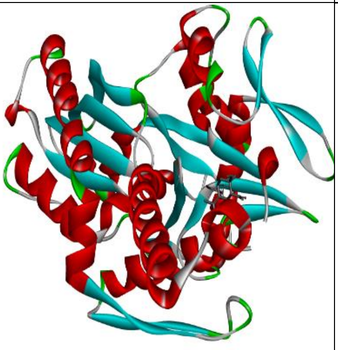	-5.3	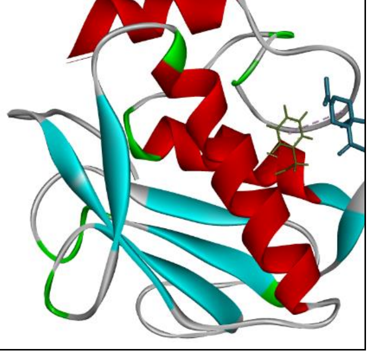

Myrcene	-4.8	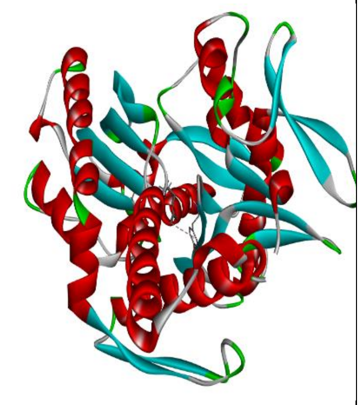	-5.2	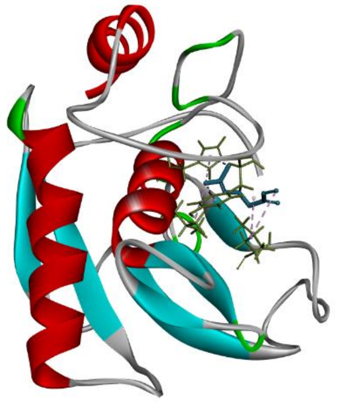
Beta-pinene	-5.4	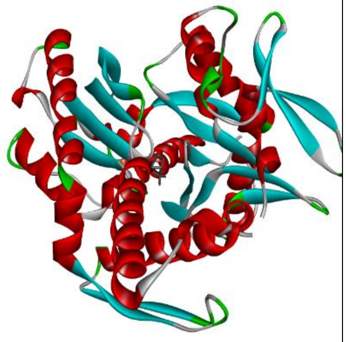	-5.2	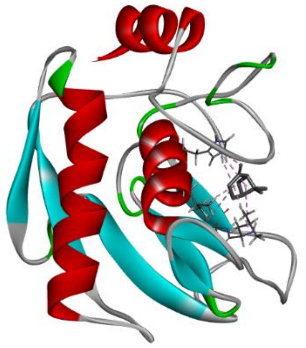
Bornyl acetate	-5.8	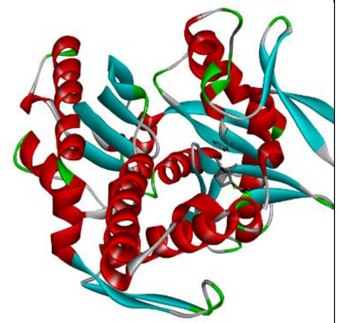	-5.1	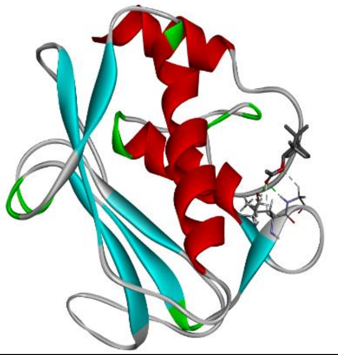
Megastigma-3,7(e),9-triene	-6.6	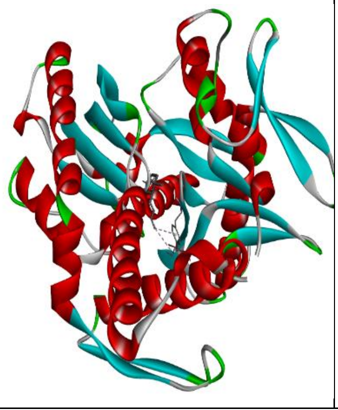	-5.1	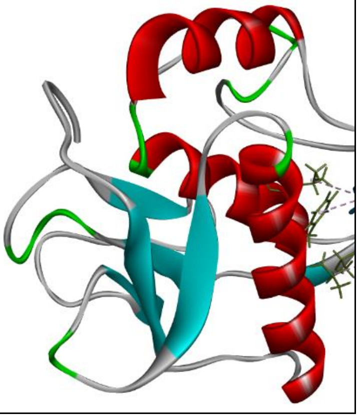

Alpha-pinene	-5.3	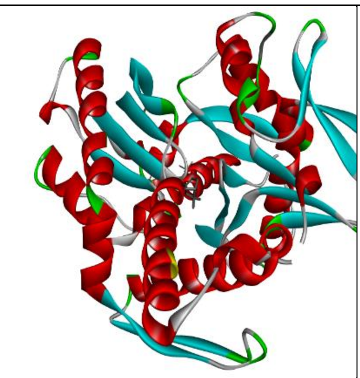	-5.1	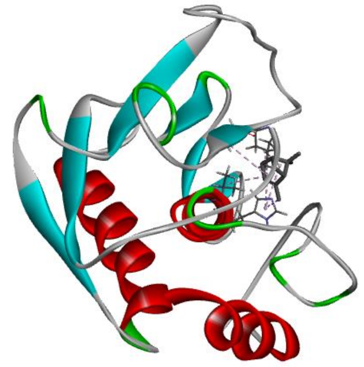
2-cyclohexen-1-one	-4.1	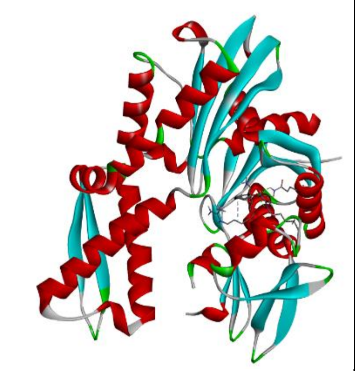	-5.0	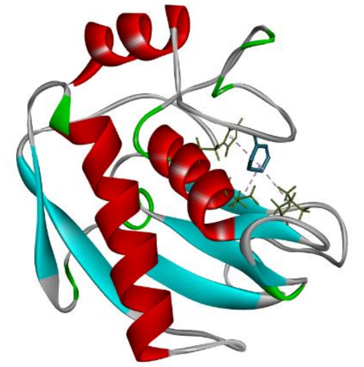
Bicyclo[3.1.0]hexane-3one	-4.5	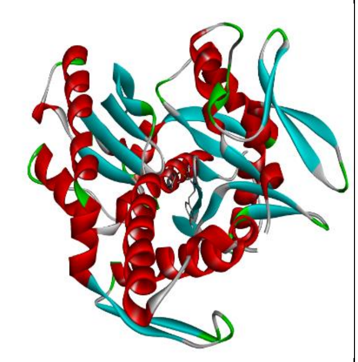	-5.0	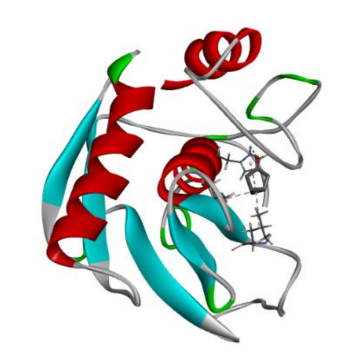
4-(dimethylamino)-,3,5-dimethyl-phenol	-5.1	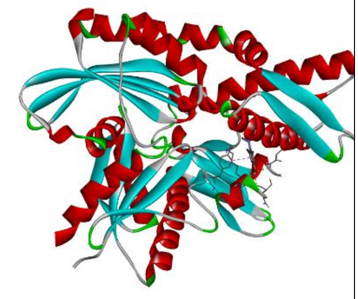	-4.9	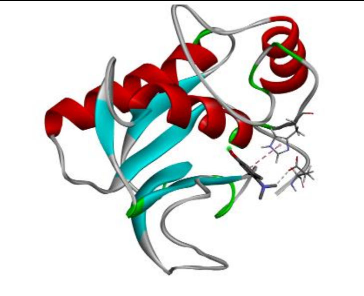
Camphor	-5.8	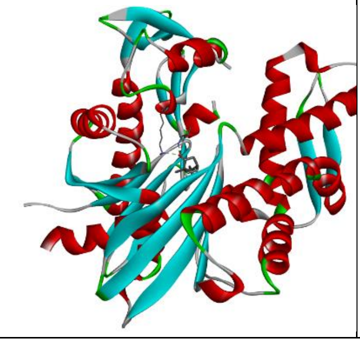	-4.9	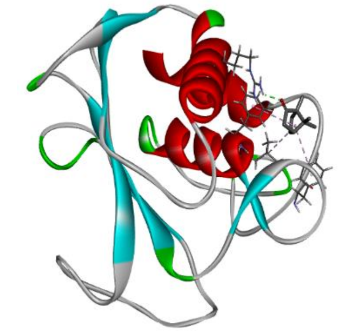

Cyclohexanol	-4.4	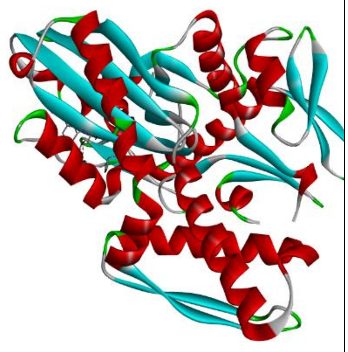	-4.9	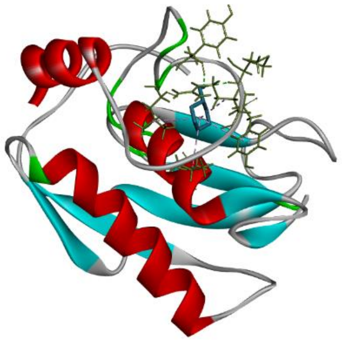
Isoborneol	-5.4	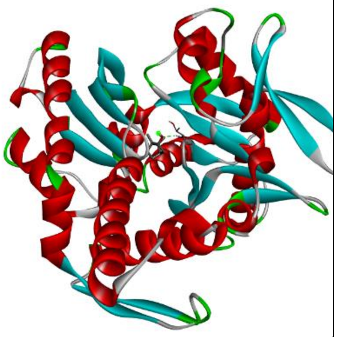	-4.9	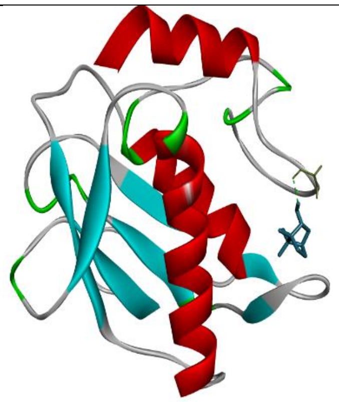
Isomenthone	-6.0	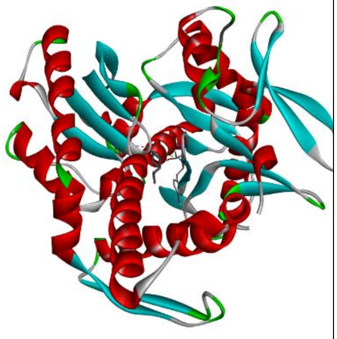	-4.9	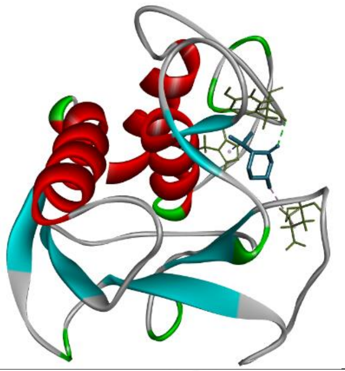
Delta-3-carene	-5.4	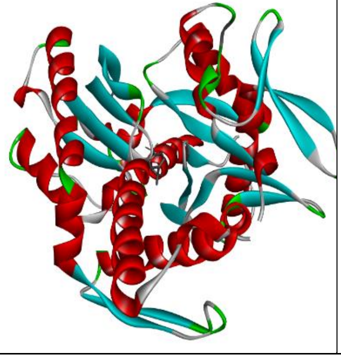	-4.9	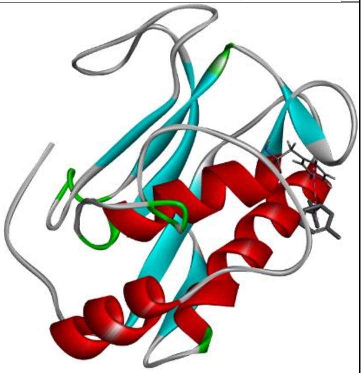

Borneol	-5.4	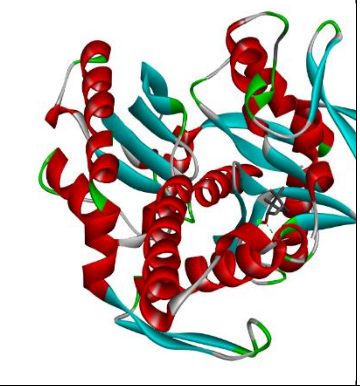	-4.7	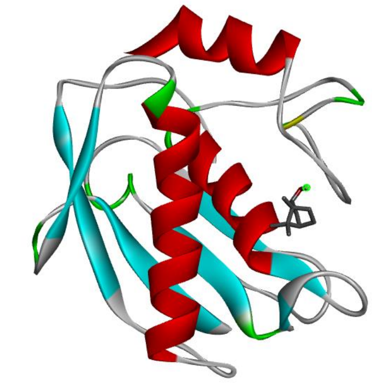
Camphene	-5.2	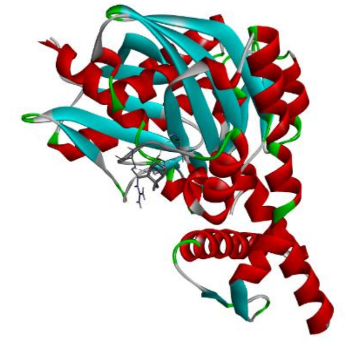	-4.7	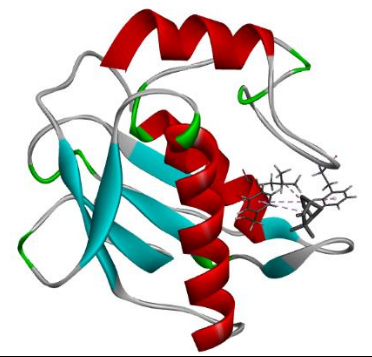
Endo-fenchol	-6.0	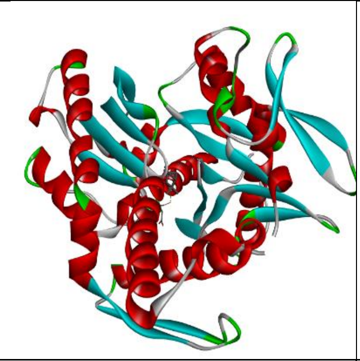	-4.7	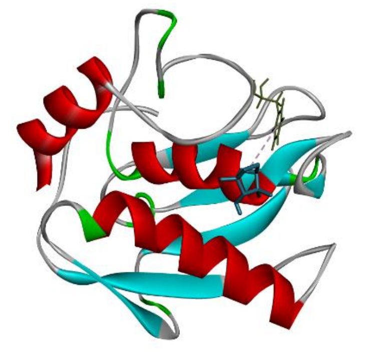
Camphene hydrate	-6.0	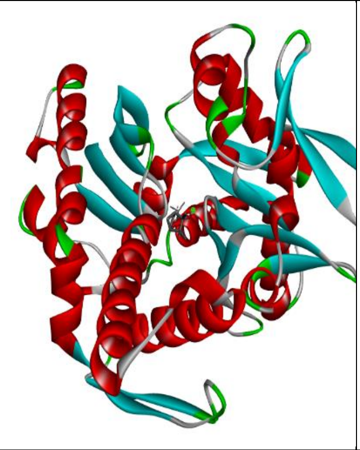	-4.6	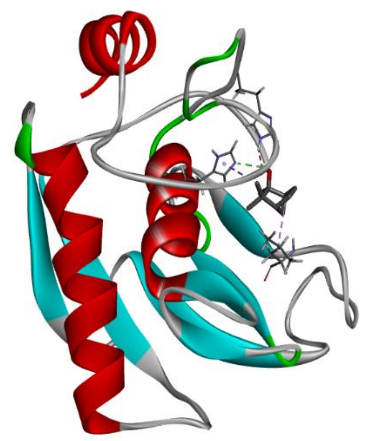

Benzene	-3.8	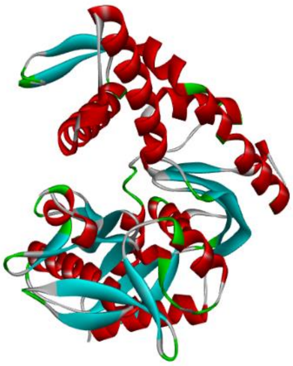	-4.5	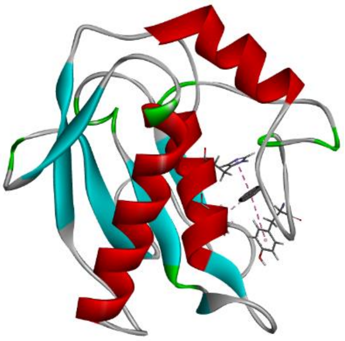
