# Supplementary material for: Targeted modulation of MMP9 and GRP78 via molecular interaction and in silico profiling of Curcuma caesia rhizome metabolites: A computational drug discovery approach for cancer therapy
Source: PLoS One. 2025 Jul 18;20(7):e0328509. doi: 10.1371/journal.pone.0328509 (PMC12273913; doi:10.1371/journal.pone.0328509)
Supplement: S4 Table — (PDF) [file pone.0328509.s004.pdf]

S4 Table: Molecular targets of curcumin and bis-demethoxycurcumin predicted from SwissTargetPrediction.

**Bis-demethoxycurcumin**	**Target**	**Common name**	**Uniprot ID**	**Probability***
	Beta-secretase 1	BACE1	P56817	0.709883036
	Monoamine oxidase A	MAOA	P21397	0.118277085
	Beta amyloid A4 protein	APP	P05067	0.118277085
	Histone acetyltransferase p300	EP300	Q09472	0.118277085
	Prostaglandin E synthase	PTGES	O14684	0.118277085
	Toll-like receptor (TLR7/TLR9)	TLR9	Q9NR96	0.118277085
	Coagulation factor VII/tissue factor	F3	P13726	0.118277085
	11-beta-hydroxysteroid dehydrogenase 1	HSD11B1	P28845	0.118277085
	DNA topoisomerase II alpha	TOP2A	P11388	0.101613855
	Tyrosine-protein kinase SRC	SRC	P12931	0.101613855
	Glyoxalase I	GLO1	Q04760	0.101613855
	Inhibitor of NF-kappa-B kinase (IKK)	IKBKG IKBKB CHUK	Q9Y6K9 O14920 O15111	0.101613855
	Phospholipase C-gamma-1	PLCG1	P19174	0.101613855
	Serine/threonine-protein kinase EEF2K	EEF2K	O00418	0.101613855
	Arachidonate 5-lipoxygenase	ALOX5	P09917	0.101613855
	Cyclin-dependent kinase 5/CDK5 activator 1	CDK5R1 CDK5	Q15078 Q00535	0.101613855
	Estradiol 17-beta-dehydrogenase 2	HSD17B2	P37059	0.101613855
	Estradiol 17-beta-dehydrogenase 1	HSD17B1	P14061	0.101613855
	Cyclin-dependent kinase 1	CDK1	P06493	0.101613855
	Serine/threonine-protein kinase Aurora-A	AURKA	O14965	0.101613855
	Serine/threonine-protein kinase mTOR	MTOR	P42345	0.101613855
	Plasminogen activator inhibitor-1	SERPINE1	P05121	0.101613855
	Glycogen synthase kinase-3 beta	GSK3B	P49841	0.101613855
	Epidermal growth factor receptor erbB1	EGFR	P00533	0.101613855
	Matrix metalloproteinase 13	MMP13	P45452	0.101613855
	Cathepsin L	CTSL	P07711	0.101613855
	Matrix metalloproteinase 8	MMP8	P22894	0.101613855
	Carbonic anhydrase VII	CA7	P43166	0.101613855
	Carbonic anhydrase I	CA1	P00915	0.101613855
	Carbonic anhydrase VI	CA6	P23280	0.101613855
	Carbonic anhydrase XIV	CA14	Q9ULX7	0.101613855
	Carbonic anhydrase IX	CA9	Q16790	0.101613855
	Carbonic anhydrase IV	CA4	P22748	0.101613855
	Carbonic anhydrase VB	CA5B	Q9Y2D0	0.101613855
	Carbonic anhydrase VA	CA5A	P35218	0.101613855
	Bone morphogenetic protein 1	BMP1	P13497	0.101613855
	Nuclear factor erythroid 2-related factor 2	NFE2L2	Q16236	0.101613855
	Cyclooxygenase-1	PTGS1	P23219	0.101613855
	Hepatocyte growth factor receptor	MET	P08581	0.101613855

	Ribosomal protein S6 kinase 1	RPS6KB1	P23443	0.101613855
	DNA topoisomerase I	TOP1	P11387	0.101613855
	Serine/threonine-protein kinase Aurora-B	AURKB	Q96GD4	0.101613855
	Serine/threonine-protein kinase RAF	RAF1	P04049	0.101613855
	Serine/threonine-protein kinase B-raf	BRAF	P15056	0.101613855
	Dynamin-1	DNM1	Q05193	0.101613855
	Tyrosine-protein kinase ABL	ABL1	P00519	0.101613855
	Platelet-derived growth factor receptor beta	PDGFRB	P09619	0.101613855
	Tyrosine-protein kinase receptor FLT3	FLT3	P36888	0.101613855
	Serine/threonine-protein kinase PIM1	PIM1	P11309	0.101613855
	Type-1 angiotensin II receptor (by homology)	AGTR1	P30556	0.101613855
	Dual specificity protein kinase CLK3 (by homology)	CLK3	P49761	0.101613855
	Serine/threonine-protein kinase Chk1	CHEK1	O14757	0.101613855
	Pyruvate dehydrogenase kinase isoform 1	PDK1	Q15118	0.101613855
	Zinc finger protein GLI2	GLI2	P10070	0.101613855
	Zinc finger protein GLI1	GLI1	P08151	0.101613855
	Serine/threonine-protein kinase WEE1	WEE1	P30291	0.101613855
	Serine/threonine-protein kinase Chk2	CHEK2	O96017	0.101613855
	Alkaline phosphatase, tissue-nonspecific isozyme	ALPL	P05186	0.101613855
	Heat shock protein HSP 90-alpha	HSP90AA1	P07900	0.101613855
	Induced myeloid leukemia cell differentiation protein Mcl-1	MCL1	Q07820	0.101613855
	Tyrosinase	TYR	P14679	0.101613855
	Multidrug resistance-associated protein 1	ABCC1	P33527	0.101613855
	Rho-associated protein kinase 2	ROCK2	O75116	0.101613855
	Dipeptidyl peptidase IV	DPP4	P27487	0.101613855
	Serine/threonine-protein kinase PLK1	PLK1	P53350	0.101613855
	Aldose reductase	AKR1B1	P15121	0.101613855
	Signal transducer and activator of transcription 6	STAT6	P42226	0.101613855
	Estrogen receptor alpha	ESR1	P03372	0.101613855
**Curcumin**	Monoamine oxidase A	MAOA	P21397	1
	Beta amyloid A4 protein	APP	P05067	1
	Histone acetyltransferase p300	EP300	Q09472	1
	Prostaglandin E synthase	PTGES	O14684	1
	Toll-like receptor (TLR7/TLR9)	TLR9	Q9NR96	1
	Beta-secretase 1	BACE1	P56817	0.807322834
	DNA topoisomerase II alpha	TOP2A	P11388	0.505302626
	Glyoxalase I	GLO1	Q04760	0.241303241
	Nuclear factor erythroid 2-related factor 2	NFE2L2	Q16236	0.217290828
	Arachidonate 5-lipoxygenase	ALOX5	P09917	0.217290828

	Cyclooxygenase-1	PTGS1	P23219	0.217290828
	Inhibitor of NF-kappa-B kinase (IKK)	IKBKG IKBKB CHUK	Q9Y6K9 O14920 O15111	0.185218735
	Epidermal growth factor receptor erbB1	EGFR	P00533	0.121287003
	Estradiol 17-beta-dehydrogenase 3	HSD17B3	P37058	0.113285953
	Signal transducer and activator of transcription 3	STAT3	P40763	0.113285953
	11-beta-hydroxysteroid dehydrogenase 1	HSD11B1	P28845	0.113285953
	Serine/threonine-protein kinase AKT	AKT1	P31749	0.113285953
	Glycogen synthase kinase-3 beta	GSK3B	P49841	0.113285953
	Carbonic anhydrase VII	CA7	P43166	0.113285953
	Carbonic anhydrase VI	CA6	P23280	0.113285953
	Carbonic anhydrase XII	CA12	O43570	0.113285953
	Carbonic anhydrase XIV	CA14	Q9ULX7	0.113285953
	Carbonic anhydrase IX	CA9	Q16790	0.113285953
	Carbonic anhydrase VA	CA5A	P35218	0.113285953
	Multidrug resistance-associated protein 1	ABCC1	P33527	0.113285953
	Serine/threonine-protein kinase Chk1	CHEK1	O14757	0.113285953
	Pyruvate dehydrogenase kinase isoform 1	PDK1	Q15118	0.113285953
	Serine/threonine-protein kinase WEE1	WEE1	P30291	0.113285953
	DNA topoisomerase I	TOP1	P11387	0.113285953
	Serine/threonine-protein kinase RAF	RAF1	P04049	0.113285953
	Serine/threonine-protein kinase B-raf	BRAF	P15056	0.113285953
	Carbonic anhydrase II	CA2	P00918	0.113285953
	Carbonic anhydrase I	CA1	P00915	0.113285953
	Glucagon receptor	GCGR	P47871	0.113285953
	Matrix metalloproteinase 14	MMP14	P50281	0.113285953
	Serine/threonine-protein kinase Aurora-B	AURKB	Q96GD4	0.113285953
	Plasminogen activator inhibitor-1	SERPINE1	P05121	0.113285953
	Ribosomal protein S6 kinase 1	RPS6KB1	P23443	0.113285953
	Serine/threonine-protein kinase Aurora-A	AURKA	O14965	0.113285953
	Cyclin-dependent kinase 2/cyclin A	CDK2 CCNA1 CCNA2	P24941 P78396 P20248	0.113285953
	Tyrosinase	TYR	P14679	0.113285953
	Type-1 angiotensin II receptor (by homology)	AGTR1	P30556	0.113285953
	NADPH oxidase 4	NOX4	Q9NPH5	0.113285953
	Bone morphogenetic protein 1	BMP1	P13497	0.113285953
	Matrix metalloproteinase 13	MMP13	P45452	0.113285953
	ADAM17	ADAM17	P78536	0.113285953
	Elastase 1	CELA1	Q9UNI1	0.113285953
	Prolyl endopeptidase	PREP	P48147	0.113285953
	Glutamate receptor ionotropic kainate 1	GRIK1	P39086	0.113285953
	Matrix metalloproteinase 8	MMP8	P22894	0.113285953

	Interleukin-8 receptor B	CXCR2	P25025	0.113285953
	Alkaline phosphatase, tissue-nonspecific isozyme	ALPL	P05186	0.113285953
	Maternal embryonic leucine zipper kinase	MELK	Q14680	0.113285953
	Inosine-5'-monophosphate dehydrogenase 1	IMPDH1	P20839	0.113285953
	Inosine-5'-monophosphate dehydrogenase 2	IMPDH2	P12268	0.113285953
	Complement factor D	CFD	P00746	0.113285953
	Sphingosine kinase 2	SPHK2	Q9NRA0	0.113285953
	Sphingosine kinase 1	SPHK1	Q9NYA1	0.113285953
	Mitogen-activated protein kinase kinase kinase 12	MAP3K12	Q12852	0.113285953
	Apoptosis regulator Bcl-2	BCL2	P10415	0.113285953
	Thyroid hormone receptor alpha	THRA	P10827	0.113285953
	Thyroid hormone receptor beta-1	THRB	P10828	0.113285953
	Macrophage colony stimulating factor receptor	CSF1R	P07333	0.113285953
	5-lipoxygenase activating protein	ALOX5AP	P20292	0.113285953
