# Supplementary material for: Targeted modulation of MMP9 and GRP78 via molecular interaction and in silico profiling of Curcuma caesia rhizome metabolites: A computational drug discovery approach for cancer therapy
Source: PLoS One. 2025 Jul 18;20(7):e0328509. doi: 10.1371/journal.pone.0328509 (PMC12273913; doi:10.1371/journal.pone.0328509)
Supplement: Attachment — Submitted filename: Reviewers comments-.docx [file pone.0328509.s005.docx]

**Manuscript Number: PONE-D-25-13435**

**Abstract**: This part is well furnished that details the outcome and findings. Yet the preceding part seems to be vague and better to keep it crispier with 2lines.

**Introduction**: Seem to be very clear about the workplan. The author should concentrate on pinpointing the importance of the study rather than explaining its importance in with vast forum, especially the first paragraph.

A complete analysis of the isolated constituents has been well explicated with detailed analysis. A distinct analysis with appropriate parameters highlights the piece of work. All the figures and tables pertaining to the methodologies are appreciable which supports the reader’s understanding.

I hereby, give my consent for the **acceptance** of the paper, after trimming the abstract and introduction contents.
